# Avocado rhizosphere community profiling: white root rot and its impact on microbial composition

**DOI:** 10.3389/fmicb.2025.1583797

**Published:** 2025-05-23

**Authors:** Phinda Magagula, Velushka Swart, Arista Fourie, Alicia Vermeulen, Johannes Harold Nelson, Zelda van Rooyen, Noëlani van den Berg

**Affiliations:** ^1^Department of Plant and Soil Sciences, University of Pretoria, Pretoria, South Africa; ^2^Hans Merensky Chair in Avocado Research, Forestry and Agricultural Biotechnology Institute (FABI), University of Pretoria, Pretoria, South Africa; ^3^Department of Biochemistry, Genetics and Microbiology, University of Pretoria, Pretoria, South Africa; ^4^Theoretical Biology and Bioinformatics, Department of Biology, Science for Life, Utrecht University, Utrecht, Netherlands; ^5^Westfalia iTeam, Westfalia Fruit, Tzaneen, South Africa

**Keywords:** *Dematophora necatrix*, metabarcoding, microbiome, soil physicochemical properties, *Persea americana*

## Abstract

**Introduction:**

The avocado rhizosphere supports diverse microbial communities essential for plant health and defence against pathogens. This study aimed to investigate the impact of *Dematophora necatrix*, the causal agent of white root rot (WRR), on the microbial composition and soil physicochemical properties of infected and non-infected avocado trees in two South African orchards.

**Methods:**

ITS and 16S metabarcoding was used to compare the composition and diversity of the rhizosphere microbiome. Soil physicochemical properties were also assessed, and culturable bacterial and fungal isolates from the rhizosphere were screened for antagonistic activity against *D. necatrix*.

**Results:**

We found that *D. necatrix* did not significantly alter overall microbial diversity but influenced relative abundance of specific taxa. In Orchard A, dominant bacterial genera included *Sphingomonas, Rokubacteriales* and *Lysobacter*, while Orchard B featured *Sphingomonas* and *Acidothermus* while beneficial microbes such as *Streptomyces* and *Bacillus* were enriched in WRR non-infected (WRR-N) soils. The fungal profiles revealed *Trichoderma* and *Penicillium* as potential biocontrol agents enriched in WRR-N soils. Furthermore, dual-culture assays demonstrated that *Bacillus, Pseudomonas, Penicillium* and *Trichoderma* isolates inhibited *D. necatrix*, highlighting their biocontrol potential. Key parameters, such as soil pH and iron (Fe), correlated strongly with microbial composition, suggesting they play an important role in pathogen resilience.

**Discussion:**

These findings underscore the complexity of the avocado rhizosphere and its role in managing WRR, offering a foundation for developing integrated disease management strategies to enhance avocado productivity.

## Introduction

*Dematophora necatrix* R. Hartig. (formerly *Rosellinia necatrix* Berl. ex Prill.) (Wittstein et al., 2020) causes white root rot (WRR) with more than 440 reported hosts (https://nt.ars-grin.gov/fungaldatabases/) (ten Hoopen and Krauss, 2006; Pliego et al., 2012; Arjona-López et al., 2024) in temperate and tropical regions globally (Petrini, 1993; Pliego et al., 2012). WRR affects several economically important fruit trees, including coffee (Castro et al., 2013), apples (Pasini et al., 2016), peaches (Dafny-Yelin et al., 2018), almonds (Palacio-Bielsa et al., 2017) and avocados (Pliego et al., 2012; van den Berg et al., 2018). WRR is a major limiting factor for avocado production in Spain (Pliego et al., 2009, 2012) and Israel (Dafny-Yelin et al., 2018). Owing to its global presence, the pathogen spread to the African continent, where it was first reported on apple trees in the Western Cape province of South Africa in the 1970s (van der Merwe and Matthee, 1974) and on avocado in the Limpopo province in 2016 (van den Berg et al., 2018). The pathogen has also been reported in Mozambique (CABI, 2024). WRR is characterized by above- and below-ground symptoms that can develop rapidly in young trees (~2–6 weeks) or gradually in older trees (~6–24 months) depending on environmental conditions. Symptoms include necrosis of the feeder roots, branch dieback, mummified fruits, yellowing and wilting of leaves, and development of white mycelial masses on roots and under the bark (Pliego et al., 2012; Granum et al., 2015). Symptoms progressively worsen leading to tree death, impacting soil health, and affecting the microbiome and physicochemical properties of the soil.

Research has shown the importance of the rhizosphere, a narrow zone of soil surrounding the roots, and endophytic microorganisms in maintaining plant health, which strongly correlates with changes in the microbial community composition in the soil (Qiao et al., 2024). Many root pathogens establish interactions with numerous microbes in the rhizosphere changing microbial diversity and richness (Chapelle et al., 2016). Pathogens like *D. necatrix*, compete with rhizosphere microbes to infect avocado trees, often producing antimicrobial effectors and toxins to suppress beneficial microbial communities which act as the first line of defense (Chavarro-Carrero et al., 2024). Previous studies in avocado, have also shown that the rhizosphere microbiome composition is significantly affected under pathogen pressure, such as *Phytophthora cinnamomi* infection (Solís-García et al., 2021; Reverchon et al., 2023). A study on citrus infected with huanglongbing disease, demonstrated that trees with slow disease progression had a higher proportion of beneficial microorganisms, while symptomatic trees were dominated by opportunistic pathogens (Ginnan et al., 2020). This shift led to an abundance of saprophytes in the advanced stages of infection. Thus, the invasion of pathogens in the rhizosphere of tree crops disrupts microbial equilibrium, resulting in dysbiosis.

The rhizosphere is further influenced by root exudates that directly affect microbial composition and diversity (Philippot et al., 2013). These exudates, including sugars, organic acids, secondary metabolites and amino acids, promote the proliferation of beneficial organisms, enhance nutrient availability, offer protection against pathogens, and sustain microbial communities essential for plant growth, health and productivity (Hinsinger et al., 2009; Hawes et al., 2012; Bulgarelli et al., 2013; Sasse et al., 2018). Previous studies have demonstrated the benefits of the rhizosphere microbiome, particularly in protecting avocado against root pathogens like *P. cinnamomi* (Solís-García et al., 2021; Reverchon et al., 2023), highlighting the importance of the microbial ecosystem in agriculture. In turn, plants devote a portion of their photosynthetically fixed carbon to nourish their associated microbial community (Santos and Olivares, 2021). Therefore, uncovering the relationship between microbial communities and plants is necessary to increase our understanding of disease development in the rhizosphere.

The composition of the rhizosphere microbial community also changes based on the time of planting, which can subsequently affect aerial tissue and the physicochemical properties of the soil (Chen et al., 2022). Such imbalances can contribute toward the aggressiveness of pathogens (Berendsen et al., 2012). The interaction between trees and the rhizosphere microbiome is unique among plants due to the long-lived perennial nature and extensive and deep root system of trees; the longevity of trees further shape the microbial community composition (Mercado-Blanco et al., 2018). Additionally, the composition and function of rhizosphere microbial communities are influenced by factors such as the plant species, cultivar, rootstock, and even the growth stage, and characteristics of the surrounding soil (Berendsen et al., 2012; Sasse et al., 2018; Castellano-Hinojosa et al., 2023). Therefore, the interactions between plants and their rhizosphere microbiomes are highly specific, underlining the need to study different host plants along with their associated microorganisms and environmental conditions.

Given the complex interactions between microbes, the host, and the variable nature of these communities this study aimed to investigate the effects of *D. necatrix* on the avocado rhizosphere. The study also confirmed the presence of *P. cinnamomi*, as this root pathogen is known to be present in all avocado growing regions and would also have an impact on the taxa relative abundance and diversity in the rhizosphere of avocado trees. We hypothesized that *D. necatrix* would change the composition and diversity of the rhizosphere, with beneficial bacteria and fungi being enriched in WRR non-infected trees. To test these hypotheses, we characterized the fungal and bacterial rhizosphere communities of WRR-infected and non-infected avocado trees through metabarcoding and soil physicochemical analyses. This study thereby provides a clearer understanding of the intricate relationship between avocado roots and their rhizosphere microbiome, as well as the effects thereon in the presence of *D. necatrix*.

## Materials and methods

### Study site selection and *D. necatrix* infection assessment

Two orchards in Tzaneen, Limpopo, South Africa (Supplementary Table 1), were previously assessed for the presence of *D. necatrix* (van den Berg et al., 2025). In short, trees were assessed and assigned a disease severity score, thereby classifying trees as either symptomatic or asymptomatic (Sherwood and Hagedorn, 1958; González-Sánchez et al., 2013). The presence of *D. necatrix* in the roots of assessed trees was determined using Taqman qPCR, species-specific primers R7 (5′-AACCATAGGCGAGATGAGAAAT-3′) and R10 (5′-CCCCTGTTGCTTAGTGTTGG-3′) (Schena et al., 2002) and the TR10-7 probe (5′-AGTCAGTGGCGGAGTCGGTC-3′) (Shishido et al., 2012). Asymptomatic trees that tested negative for the presence of *D. necatrix* were reclassified as uninfected. Therefore, trees in both orchards were assigned as either WRR-S (symptomatic, *D. necatrix* infected), WRR-AS (asymptomatic, *D. necatrix* infected) or WRR-N (asymptomatic, non-infected).

### Rhizosphere soil, rootlets, and feeder roots sample collection

Rhizosphere soil rootlets and feeder root samples were collected from 30 avocado trees in each orchard (Orchards A and B) as described by Guevara-Avendaño et al. (2018). For each tree, approximately 5–20 g soil was collected around the tree, targeting areas where some feeder roots were present. Samples were homogenized to create a single rhizosphere soil sample for each tree. We collected 60 soil samples including feeder roots (10 trees per category per orchard; WRR-S, WRR-AS and WRR-N), samples were kept at 4°C prior to DNA extraction.

### Confirmation of the presence of *P. cinnamomi* in orchard A and B

Rhizosphere soils, rootlets, and feeder root samples were collected for *P. cinnamomi* detection from both avocado orchards in similar trees (30 avocado trees per orchard) used for the detection of *D. necatrix* and metabarcoding analysis. Rootlets and feeder roots isolations were made by embedding root pieces in NARPH selective media (20% clarified V8 juice agar containing 50 mg/l nystatin, 200 mg/l ampicillin, 10 mg/l rifampicin, 20 mg/l pentachloronitrobenzene, 50 mg/l hymexazol, and 20 g/l agar) and incubated at 25°C for 3–4 days. Rhizosphere soil samples were prepared for detection of *P. cinnamomi* using the leaf disc baiting method (Sena et al., 2018). For each sample a 40 ml aliquot of soil was placed in a sterile 50-ml cup, flooded with sterile water, and baited with 5–10 avocado leaf disks (~5 mm in diameter) punched from surface-sterilized (wiped with 70% ethanol) leaves. Cups were incubated in a dark growth chamber at 25°C for 7 days. Following incubation, three leaf disks were placed on two plates containing NARPH selective media and incubated at 25°C for 3–4 days. For further analysis, NARPH cultures were transferred to ½ potato dextrose agar (PDA; 19.5 g/l PDA agar and 10 g/l agar) by excising a block of media where only one organism was growing or selectively transferring the *P. cinnamomi* mycelia (white fluffy mycelia) to fresh ½ PDA agar plates.

Cultures were incubated, in the dark, at 25°C for 2–3 days and used to excise single hyphal tips. Single hyphal tips were cultured on 2% malt extract agar (MEA; 20 g/l malt extract and 15 g/l agar). A mycelial mass was obtained, freeze-dried, and stored at −80°C. Genomic DNA was extracted from the freeze-dried mycelia using PrepMan Ultra Sample Preparation Reagent (Thermo Fisher Scientific, Waltham, USA) as described by Engelbrecht et al. (2017). The identity of isolates was confirmed by PCR using species-specific primers that amplify the *Ypt1* gene (Ycin3F, 5′-GTCCTATTCGCCTGTTGGAA-3′; and Ycin4R, 5′-GGTTTTCTCTACATAACCATCCTATAA-3′) (Schena et al., 2008).

## ITS and 16S rRNA metabarcoding

### DNA extraction and PCR and sequencing

For metabarcoding, genomic DNA was extracted from the soil using the NucleoSpin^®^ Soil DNA extraction kit (Macherey-Nagel, Düren, Germany), according to the manufacturer's instructions. DNA concentration and purity was assessed using a NanoDrop ND2000C spectrophotometer (Thermo Fisher Scientific, Waltham, USA), visualized on a 1.5% agarose gel, and stored at −20°C until further analysis. The presence of *D. necatrix* in the DNA extracted from the soil samples was confirmed as previously described.

For microbial community analysis, genomic DNA was sequenced by Inqaba Biotechnical Industries (Pty) Ltd. (Pretoria, South Africa) using PacBio Sequel IIe platform. Bacterial *16S rRNA* were amplified using 27F_PB (5′-AGRGTTYGATYMTGGCTCAG-3′) and 1492R_PB (5′-RGYTACCTTGTTACGACTT-3′) primers. The fungal internal transcribed spacer (ITS) region was amplified using the ITS1F_PB (5′-CTTGGTCATTTAGAGGAAGTAA-3′) and ITS4_PB (5′-TCCTCCGCTTATTGATATGC-3′) primers. Barcoding and library preparation was conducted according to the manufacturer's procedures prior to sequencing (Pacific Biosciences^®^, Menlo Park, USA).

## ITS and 16S rRNA metabarcoding read processing and generation of amplicon variants

### Preprocessing and annotation of sequence reads

Longer reads resulted in too much variation to reliably identify amplicon sequence variants (ASVs); thus, the reads were trimmed using Cutadapt (Martin, 2011) to the same amplicon length as used in Illumina metabarcoding protocols. The primer sequences used for trimming the ITS region were ITS1 (5′-TCCGTAGGTGAACCTGCGG-3′) and ITS4 (5′-TCCTCCGCTTATTGATATGC-3′) (Solís-García et al., 2021). The *16S rRNA* sequences were trimmed using F341 (5′-CCTACGGGNGGCWGCAG-3′) and R805 (5′-GACTACHVGGGTATCTAATCC-3′) primers (Herlemann et al., 2011; Qiu et al., 2020). Reads were filtered, trimmed, and denoised using DADA2 v.1.26.0 (Callahan et al., 2016), setting the parameters minLen = 400, maxLen = 600, maxN = 0, maxEE = 2, and minQ = 2. The reads were then merged, and chimeras (between 6,000 and 40,000 per sample) were removed using DADA2 to obtain ASVs. Taxonomic assignment was performed using the SILVA SSU r138.1 reference database for bacterial taxa (Pruesse et al., 2007; Quast et al., 2013), and the Unite database v10.5.2021 (Nilsson et al., 2019) for fungal taxa using the naïve Bayes classifier in QIIME2 (Bolyen et al., 2019). ASVs with very low read numbers (average relative abundance <1^e − 5^) were excluded from the dataset using phyloseq v. 3.19 (Mcmurdie and Holmes, 2013). The ASVs abundance tables and taxonomic classification output files from the DADA2 pipeline were used for downstream data exploration, statistical analysis, and visualization. ASVs filtering removed archaeal, eukaryotic, chloroplastic, and mitochondrial reads from the bacterial and fungal datasets.

## Analysis of the rhizosphere microbiome using 16S rRNA and ITS metabarcoding

### Fungal and bacterial community composition of healthy and WRR infected avocado trees

Following taxonomic classification, community composition was investigated at the genus level, clustering ASVs with a relative abundance > 1^e − 5^, using the phyloseq package (Mcmurdie and Holmes, 2013). Genera with a relative abundance >0.6% were selected to construct a bar graph displaying abundance patterns across the symptomatic (WRR-S), asymptomatic (WRR-AS), and non-infected (WRR-N) datasets, utilizing ggplot2 (Wickham, 2016).

### Diversity analysis of bacterial and fungal communities of the avocado rhizosphere

Richness and evenness (alpha diversity) were determined using Shannon's diversity index (emphasizing species richness), Simpson's index (focussing on evenness), and Chao1 diversity index (for species richness). Significant differences in the alpha diversity across different populations were tested using the Kruskal-Wallis test; *p* < 0.05, followed by a Wilcoxon rank-sum test; *p* < 0.05, for pairwise comparisons. The Benjamini-Hochberg method was used to adjust the *p*-values for multiple comparisons.

Dissimilarities between communities were evaluated based on Bray-Curtis dissimilarity metrices, which consider both composition and abundance of ASVs. Data dispersion homogeneity within each group was confirmed using betadisper, followed by permutational multivariate analysis of variance; *p* > 0.05, using the Adonis function in the vegan package v.2.6-4 (Oksanen et al., 2022). Pairwise comparisons to identify significantly different populations were performed using the pairwise Adonis package, with *p*-values adjusted using the Benjamini-Hochberg method (Martínez Arbizu, 2020). In addition, distances were used to construct a non-metric multidimensional scaling ordination plot to visualize the clustering of individuals from all populations. All statistical analyses were performed with R v.4.0.3 (R Core Team, 2021) in RStudio (RStudio Team, 2021).

### Enrichment of specific bacterial and fungal genera

We investigated the enrichment of fungal and bacterial genera among WRR-S, WRR-AS, and WRR-N categories. We employed DESeq2 v 1.30.1 (Love et al., 2014) in RStudio (RStudio Team, 2021), to identify genera that were significantly enriched. Results were filtered using an adjusted *p*-value (*padj*) of ≤ 0.05 and a log2 fold change of ±1. Genera were further filtered with Benjamini-Hochberg-corrected *p*-values < 0.001 in RStudio (RStudio Team, 2021).

### Unique bacterial and fungal genera between WRR infected and non-infected avocado trees

We investigated unique genera within WRR-S, WRR-AS, and WRR-N categories. We identified bacterial and fungal genera with a relative abundance of 0.001. For analyses and visualization of intersections, UpSet plot graphs were created in RStudio (RStudio Team, 2021) using the UpSetR package v. 1.4.0 (Conway et al., 2017).

## Rhizosphere soil physicochemical properties of two avocado orchards

Soil samples were collected from Orchards A and B, specifically targeting the rhizosphere soil around the tree trunk. Ten replicated samples were collected for each of the groups (WRR-S, WRR-AS and WRR-N) in two orchards. Rhizosphere soil physicochemical properties were analyzed at the Department of Plant and Soil Sciences Laboratory, University of Pretoria, South Africa. Details of the analyses used were outlined previously (van den Berg et al., 2025). Data were subjected to one way ANOVA and Tukey's test to determine significant differences at *p* ≤ 0.05.

## The relationship between rhizosphere soil physicochemical properties and microbial community composition

The physicochemical properties and microbial communities of the rhizosphere soil were analyzed using principal component analysis (PCA) with R v.4.0.3 (R Core Team, 2021) in RStudio (RStudio Team, 2021). Variables representing physicochemical properties were included as meta-variables to explore their interactions with microbial communities. To perfom the PCA analyses, we selected the top (0.6%) fungal and bacterial genera from each category of trees in both orchards.

## Assessing the *in vitro* antagonistic effect of fungi and bacteria isolated from the avocado rhizosphere

### Isolation of culturable fungi and bacteria from rhizosphere soil

Rhizosphere soil samples obtained from WRR-N trees in Orchard B were used to isolate culturable fungi and bacteria. Bacteria and fungi were initially isolated using a serial dilution. Fungi were isolated by diluting soil samples up to 10^−3^, plating on ½ PDA with antibiotics (Ampicillin (200 μl/ml), Rifampicin (10 μl/ml) and Streptomycin (50 μl/ml)) and incubating at 25°C in the dark until growth appeared. Pure fungal cultures were obtained by transferring hyphal tips from the edges of colonies to fresh PDA. Fungal isolates were categorized into morphotypes based on macroscopic and microscopic features, with conidial shape, color, and size characterized using 25 conidia per isolate. To isolate bacteria, soil samples were diluted up to 10^−5^ and plated on Luria-Bertani (LB) medium (10 g/l tryptone, 5 g/l yeast extract, and 5 g/l NaCl). The plates were incubated in the dark at 25°C for 1–2 days. Pure cultures were obtained by selecting single colonies and streaking them on fresh LB agar plates.

### Screening and determination of antagonistic effect of culturable bacteria and fungi

Initially, approximately 60 fungal and 100 bacterial isolates were screened *in vitro* for their ability to inhibit *D. necatrix* growth using the isolate ARP-2017-Rn2, a South African isolate previously shown to be highly virulent on avocado (van den Berg et al., 2018). Those isolates that showed some levels of inhibition (20 fungal and 22 bacterial isolates) were selected for further testing using dual-culture assays. The *D. necatrix* isolate was grown on ½ PDA at 25°C in the dark for 7 days. For fungal dual-culture assays, a 5-mm agar plug from a 5-day-old fungal culture was placed opposite a 5-mm plug from a 7-day-old *D. necatrix* culture (20 mm from the plate periphery) on a 90-mm Petri dish containing ½ PDA. For bacterial isolates, a 5-mm agar plug from a 7-day-old *D. necatrix* culture was placed in the center of each plate, and single bacterial colonies from 5-day-old cultures were positioned at four equidistant locations, 20 mm from the periphery. Each fungal and bacterial isolate was tested on a separate plate, with three replicates each. Control plates contained only *D. necatrix*. All plates were incubated at 25°C in the dark for 8 days, and the radial growth of *D. necatrix* was measured. The percentage inhibition (PI) of mycelial growth was calculated using Equation 1, where *C* is the diameter (mm) of the radial growth of *D. necatrix* in the control, and *T* is the diameter (mm) of the radial growth of *D. necatrix* in the presence of antagonistic strains. All analyses were performed in R v.4.0.3 (R Core Team, 2021) using RStudio (RStudio Team, 2021).


(1) $$\begin{array}{l} PI=100\;\times \frac{C-T}{C} \end{array}$$

### Molecular identification of bacterial and fungal isolates

Fungi and bacteria were identified by sequencing the ITS and 16S rRNA regions, respectively. DNA was extracted from one fungal isolate per morphotype (*n* = 10) using the CTAB protocol (Brunner et al., 2001). For bacteria, a single colony was added to 50 μl CTAB without grinding. Sequencing was performed using specific primers: fungal ITS sequencing using ITS1 and ITS4 primers (Solís-García et al., 2021), and bacterial 16S rRNA amplification using F341 and R805 primers (Herlemann et al., 2011; Qiu et al., 2020). The EmeraldAmp^®^ GT PCR Master Mix (Takara Bio Inc., Kusatsu, Japan) was used with the following cycling conditions: 98°C for 1 min, 35 cycles at 98°C for 10 s, 60°C for 30 s, 72°C for 1 min, followed by 72°C for 1 min. Amplicons were purified using the EXO-SAP protocol and sequenced on an ABI 3500xl genetic analyser (Thermo Fisher Scientific, Waltham, USA).

Sequences were trimmed and consensus sequences were constructed using the CLC Main Workbench 22.0.1 (QIAGEN, Hilden, Germany). These sequences were then identified through Basic Local Alignment Search Tool (BLAST) analysis. This step involved comparing the obtained sequences to known sequences in NCBI GenBank (Sayers et al., 2020) to determine the species of fungi and bacteria. Data from these analyses, were compiled and visualized using R v.4.0.3 (R Core Team, 2021) and RStudio (RStudio Team, 2021).

## Results

### Confirmation of the presence of *D. necatrix* and *P. cinnamomi* in two avocado orchards

The qPCR analysis confirmed the presence of *D. necatrix* in infected samples. Based on these data, rhizosphere soil and root samples from 30 trees in both Orchards A and B were grouped into three categories: WRR-S (symptomatic and *D. necatrix* infected), WRR-AS (asymptomatic and *D. necatrix* infected), or WRR-N (asymptomatic and non-infected) with each category comprising of 10 trees per orchard (Table 1, Supplementary Table 2). As expected, *P. cinnamomi* was present in all 60 trees across the two orchards.

**Table 1:** Infection status of trees based on the detection of the root rot pathogens *Phytophthora cinnamomi* and *Dematophora necatrix*.

**Orchard**	**Category**	**No. of samples**	**Confirmation of *P*.*cinnamomi*^a^**	**Confirmation of *D*.*necatrix*^b^**
			**Baiting** + **NARPH**	**qPCR detection**
Orchard A	WRR-S	10	Positive	Positive
	WRR-AS	10	Positive	Positive
	WRR-N	10	Positive	Negative
Orchard B	WRR-S	10	Positive	Positive
	WRR-AS	10	Positive	Positive
	WRR-N	10	Positive	Negative

^a^Soil samples were used to bait for *P. cinnamomi*.

^b^DNA isolated from the tree roots were screened using the *D. necatrix* specific qPCR assay.

### Diversity analysis of bacterial and fungal communities in rhizosphere soil from two avocado orchards

Following quality filtering, trimming and chimera removal, 8 310 bacterial and 1 919 fungal ASVs were obtained from Orchard A, and 9 656 bacterial and 1 771 fungal ASVs were obtained from Orchard B (Table 2). Sequences were submitted to the GenBank NCBI database under BioProject PRJNA1149920 (https://www.ncbi.nlm.nih.gov/bioproject/).

**Table 2:** Summary of sequencing data from avocado tree rhizosphere soil samples.

**Organisms**	**Orchard**	**Raw sequences**	**High-quality sequences**	**Amplicon sequence variants (ASVs)**
Bacteria	A	464,397	291 628	8,310
	B	552,871	342,827	9,656
Fungi	A	356,783	340,147	1,919
	B	393,207	360,103	1,771

**Figure 1:**
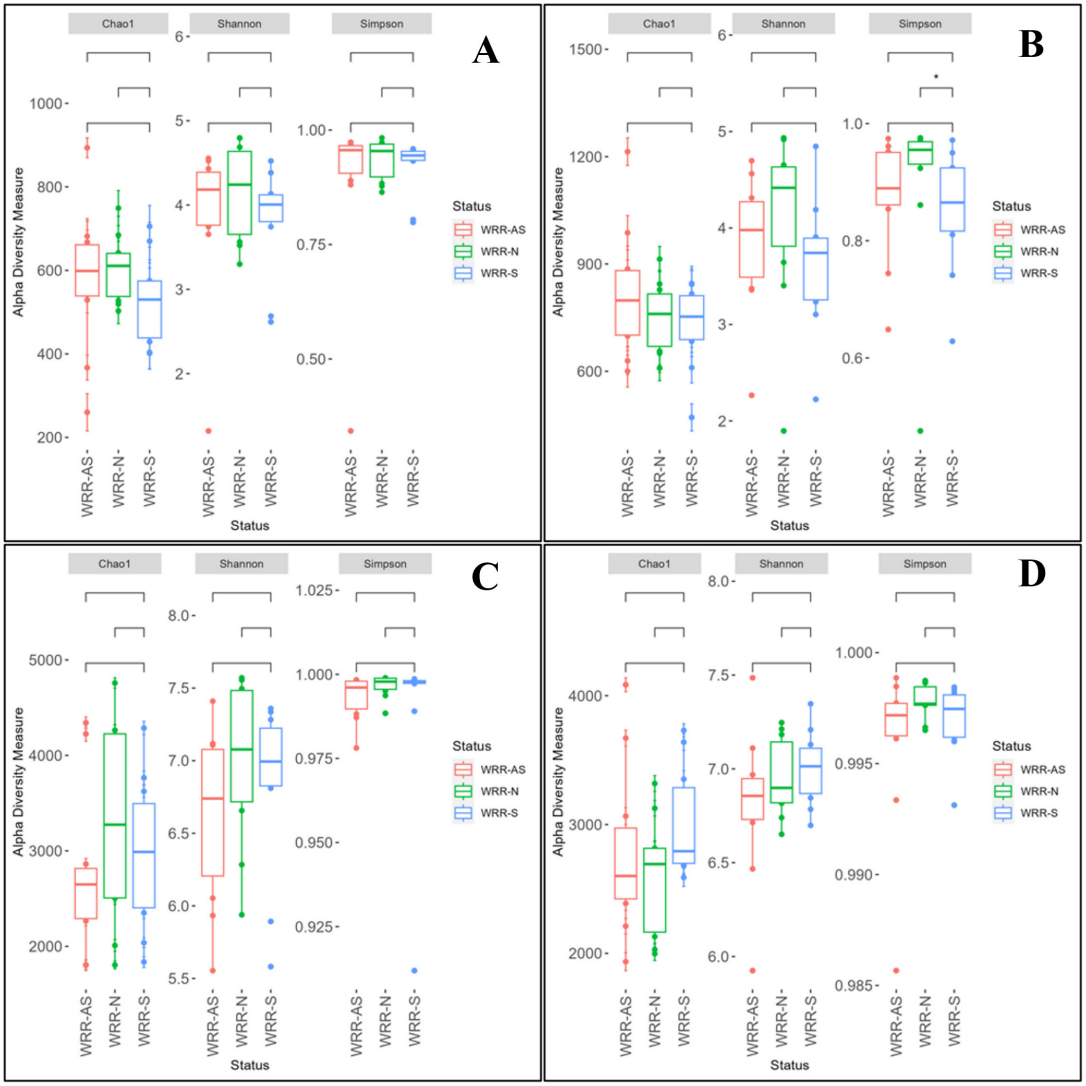
Alpha diversity of the microbiome of bacterial and fungal communities in the avocado rhizosphere. Samples were collected from soil around white root rot asymptomatic (WRR-AS), white root rot non-infected (WRR-N), and white root rot symptomatic (WRR-S) trees. The graphs represent alpha diversity indices for Chao1 diversity, Shannon diversity, and Simpson diversity of bacteria in Orchard A **(A)** and Orchard B **(B)**; and fungi in Orchard A **(C)** and Orchard B **(D)**. Horizontal bars within boxes represent the median. The top and bottom of boxes represent 75^th^ and 25^th^ quartiles, respectively. The upper and lower whiskers represent the range of non-outlier data values. The outliers were plotted as individual points. The star indicated significant differences among respective groups based on two-sided tests by Wilcoxon rank-sum test followed by Tukey multiple comparison test (Benjamini–Hochberg method adjusted *p* < 0.05).

Alpha diversity indices (Chao1, Shannon, and Simpson; Figure 1) for bacterial and fungal communities in soil samples from Orchards A and B showed no significant differences across WRR-N, WRR-AS, and WRR-S categories, with one exception: in Orchard B, the bacterial Simpson index differed significantly between WRR-N and WRR-S (Figure 1B). The WRR-N trees demonstrated lower bacterial diversity than what was observed for WRR-S trees. Beta diversity, assessed using Bray-Curtis distances, also revealed no significant differences or clustering between bacterial and fungal communities across categories in either orchard (Supplementary Figure 1). Overall, the presence of *D. necatrix* and *P. cinnamomi* did not significantly impact microbial community diversity in the avocado rhizosphere.

### Bacterial community composition of two avocado orchards

**Figure 2:**
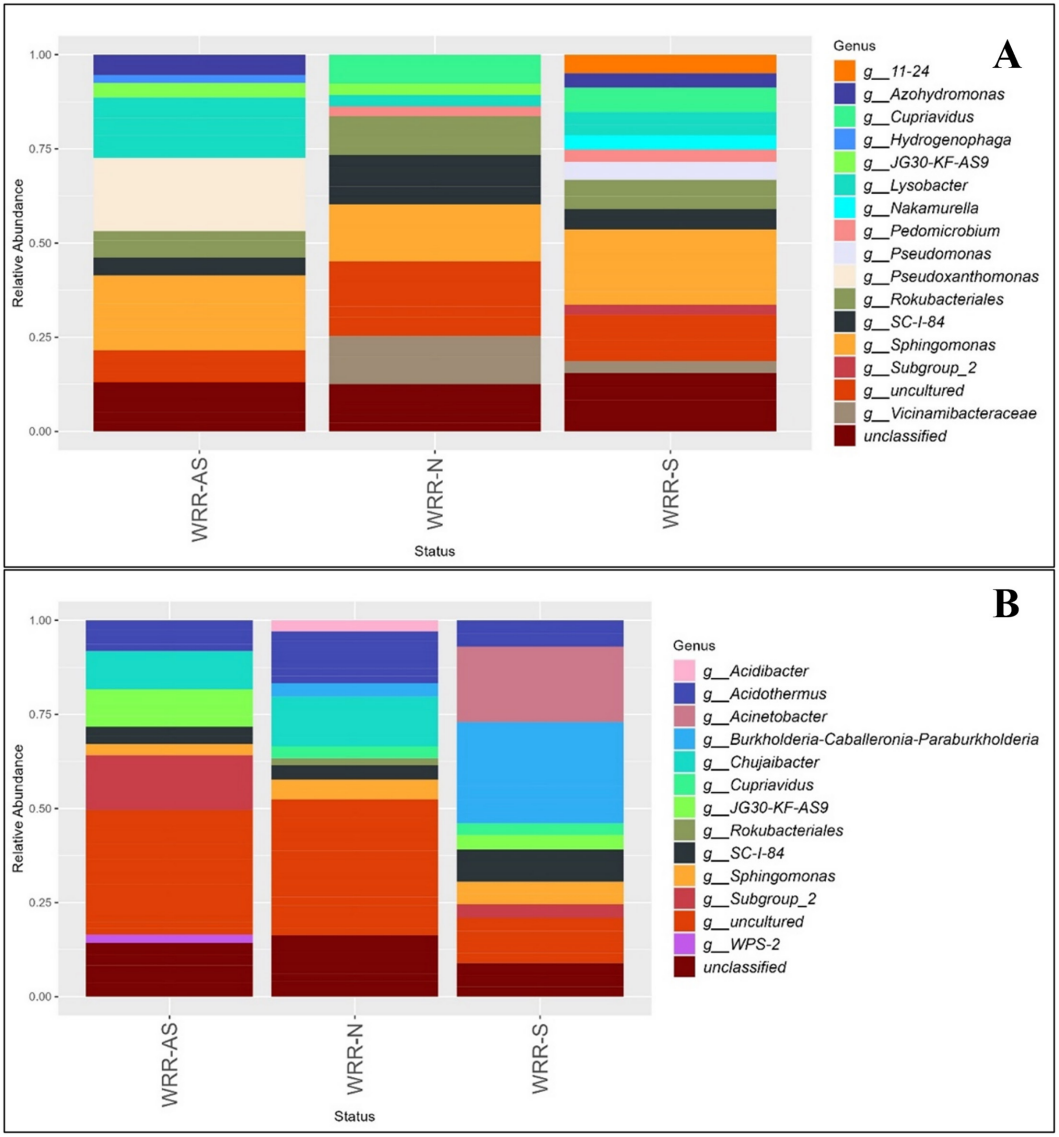
Relative abundance bar chart of the most abundant genera of the bacterial community in the rhizosphere of samples collected in **(A)** Orchard A and **(B)** Orchard B. Samples were collected from soil around white root rot asymptomatic (WRR-AS), white root rot non-infected (WRR-N), and white root rot symptomatic (WRR-S) trees. The most dominant genera above 0.6% were identified using 16S metabarcoding data. Unclassified indicates the proportion of ASVs which could not be described to genus level.

The community composition of bacteria in both orchards at phylum level was dominated by *Actinobacteria, Acidobacteria, Chloroflexi, Gemmatimonadota, Proteobacteria*, and *Plactomycetota* (Supplementary Figure 2). The bacterial communities exhibited similar dominant genera (>0.6%) across the WRR-S, WRR-AS, and WRR-N categories in both Orchard A and B (Figure 2). However, the number of ASVs across bacterial taxonomic classifications (phyla, class, order, family, and genus) varied between the two orchards (Supplementary Table 3).

In Orchard A, *Sphingomonas, Rokubacteriales, SC-I-84* and *Lysobacter* were abundant across all three tree categories (Figure 2A). *Pseudoxanthomonas* was the most prominent genus in the WRR-AS samples, but did not feature amongst the most dominant genera in either WRR-N or WRR-S. WRR-S samples featured *Pseudomonas, Nakamurella, Subgroup_2* and *11-24*, which were not amongst the most dominant genera in either WRR-AS or WRR-N samples. *Pedomicrobium* was one of the most dominant genera in WRR-N and WRR-S but did not feature in WRR-AS samples.

In Orchard B, *Sphingomonas, SC-I-84*, and *Acidothermus* was amongst the most dominant genera across all categories (Figure 2B). WRR-AS samples were dominated by genera *Subgroup_2* and *Chujaibacter*, as well as *WPS-2* which was not dominant in WRR-N or WRR-S. In contrast, the WRR-S sample profiles featured *Acinetobacter* and *Burkholderia-Caballeronia-Paraburkholderia* (*BCP*) as the most prominent genera. *BCP* was previously classified under *Burkholderia*, but has now been reclassified into separate genera.

In both orchards, uncultured and unclassified genera were present across all categories. WRR-N samples from both orchards shared similar dominant genera, while WRR-S and WRR-AS samples displayed more distinct bacterial community compositions.

### Fungal community composition of two avocado orchards

**Figure 3:**
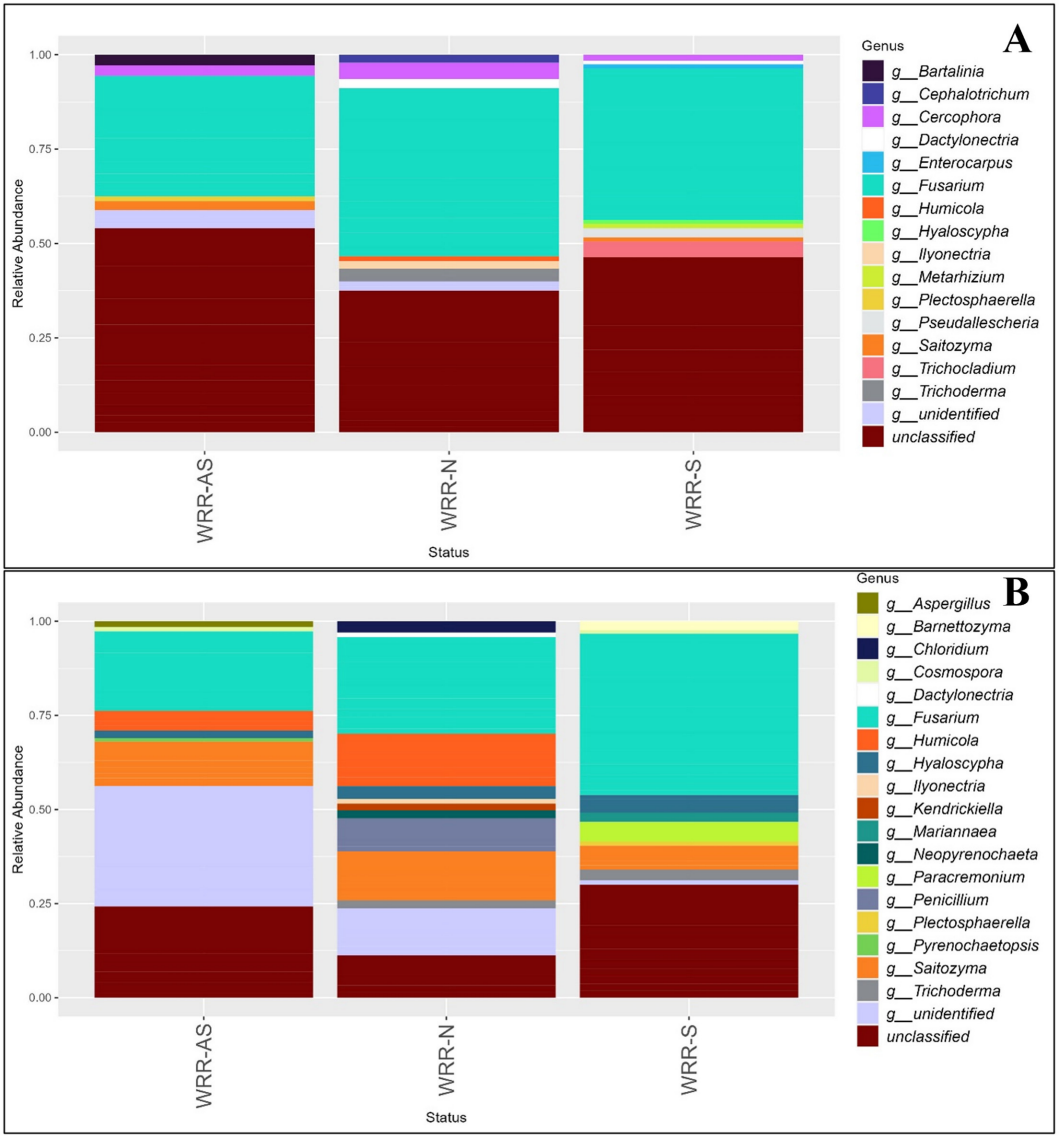
Relative abundance bar chart of the genera of the fungal community in the rhizosphere of samples collected in **(A)** Orchard A and **(B)** Orchard B. Samples were collected from soil around white root rot asymptomatic (WRR-AS), white root rot non-infected (WRR-N), and white root rot symptomatic (WRR-S) trees. The most dominant genera above 0.6% were identified using ITS metabarcoding data. Unclassified indicates the proportion of ASVs which could not be described to genus level.

The community composition of fungi at the phylum level in both orchards was dominated by *Basidiomycota, Ascomycota*, and *Rozellomycota* (Supplementary Figure 3). Fungal communities showed no significant shifts in dominant genera (>0.6%) across the WRR-S, WRR-AS and WRR-N categories (Figure 3). However, the number of ASVs varied across fungal taxonomic groups between the orchards (Supplementary Table 3).

In Orchard A, *Fusarium* and *Cercophora* were found amongst the dominant genera across all categories, with *Fusarium* being the most dominant (Figure 3A). *Trichoderma, Ilyonectria, Humicola* and *Cephalotrichum* featured among the dominant genera in WRR-N, but not in the WRR-AS and WRR-S samples. Unique dominant genera which featured in the WRR-S samples included *Trichocladium, Metarhizium, Enterocarpus* and *Pseudallescheria;* while WRR-AS featured *Bartalinia. Dactylonectria* was dominant genera in both WRR-N and WRR-S samples.

Again *Fusarium* was the dominant genus in all three tree categories from Orchard B, with *Saitozyma* and *Hyaloscypha* also featuring across all categories (Figure 3B). Notably, *Penicillium* and *Chloridium* were among the dominant genera in the WRR-N category, but were not dominant in the WRR infected categories. WRR-S samples featured *Barnettozyma, Mariannaea, Cosmospora, Plectosphaerella* and *Paracremonium;* but *Humicola* did not feature among the dominant genera as it did in WRR-AS and WRR-N. The WRR-N samples showed the greatest fungal diversity among the dominant genera, with *Penicillium, Neopyrenochaeta, Kendrickiella, Illyonectria* and *Dactylonectria* being dominant in WRR-N only.

### Enrichment of specific bacterial and fungal genera in two avocado orchards

**Figure 4:**
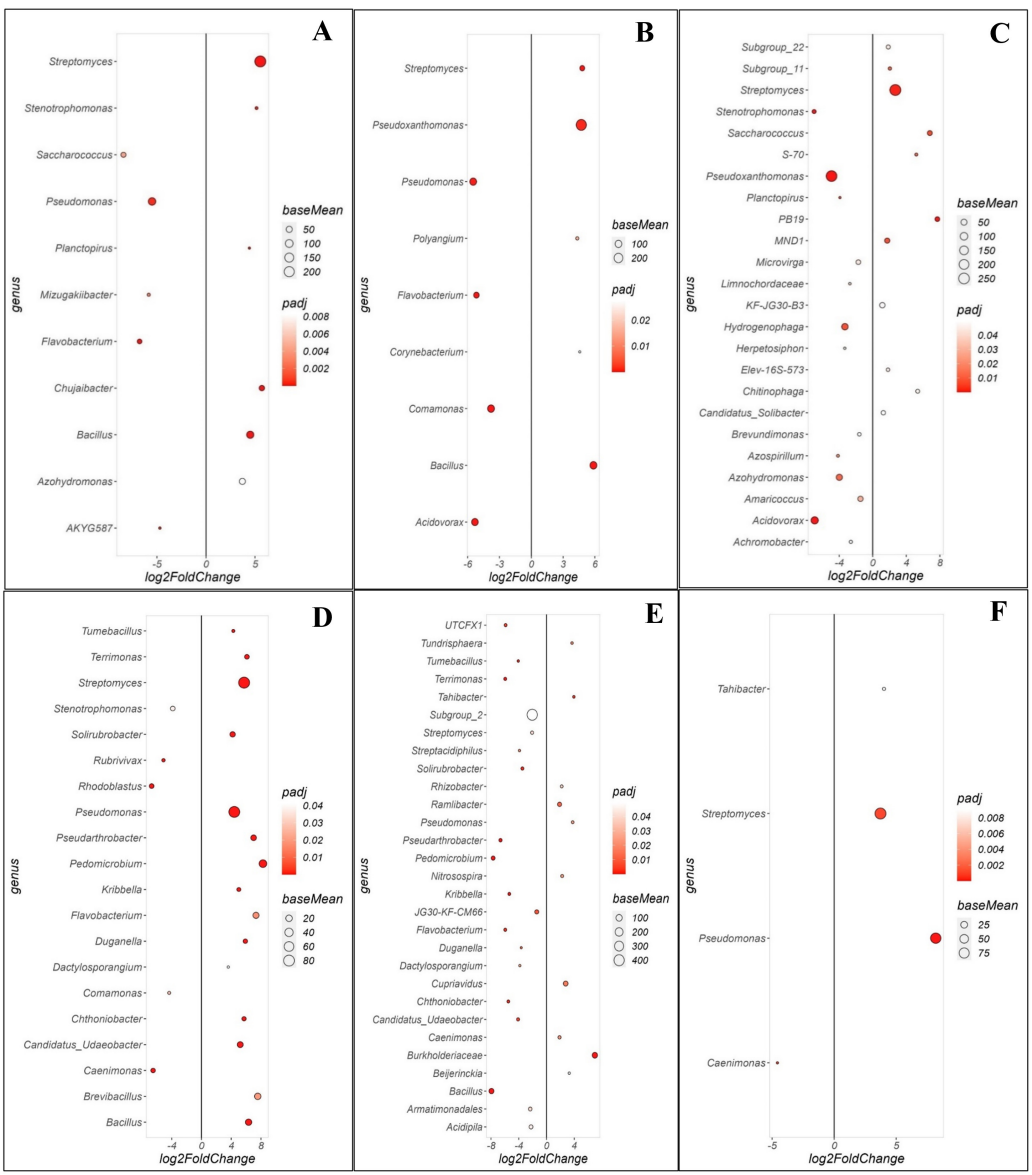
Comparison of specific bacterial genera that were either enriched or depleted between different groups of soil samples collected in the rhizosphere in Orchards A and B. Comparison of rhizosphere soil samples between **(A)** WRR-S vs. WRR-N, **(B)** WRR-S vs. WRR-AS, **(C)** WRR-AS vs. WRR-N from Orchard A and **(D)** WRR-S vs. WRR-N, **(E)** WRR-S vs. WRR-AS, **(F)** WRR-AS vs. WRR-N from Orchard B. The group mentioned first was used as a reference when determining statistical differences; in the figure it will always have a positive log2fold change and thus be enriched in the reference group. The fold-change is shown on the X-axis and the genera are listed on the Y-axis. Each colored dot represents a genus population that is significantly more or less abundant (*p* ≤ 0.05). The size of the circle shows the average abundance, and the color intensity of the circle shows *padj* value (significance level). A larger circle indicates higher relative abundance in each population.

Bacterial genera in both orchards exhibited distinct enrichment patterns when comparing communities between the WRR-S, WRR-AS and WRR-N trees (Figure 4). In Orchard A, a comparison between WRR-S and WRR-N revealed 11 significant genera (Figure 4A). WRR-S was notably enriched in *Pseudomonas*, in high relative abundance, as well as *Flavobacterium* and *Saccharococcus* both present in lower relative abundance. In contrast, WRR-N samples showed enrichment for *Streptomyces, Bacillus* and *Chujaibacter*. When comparing WRR-S and WRR-AS, nine significant genera were observed (Figure 4B). WRR-S samples were enriched with *Acidovorax, Pseudomonas, Comamonas*, and *Flavobacterium*; while WRR-AS samples were enriched with *Pseudoxanthomonas, Bacillus* and *Streptomyces*. A comparison of WRR-AS and WRR-N revealed 24 significant genera (Figure 4C). WRR-AS samples were enriched for *Pseudoxanthomas, Acidovorax, Azohydromonas, Hydrogenophaga* and *Stenotrophomonas*; while WRR-N was enriched with *Streptomyces* which also showed a high relative abundance compared to *Saccharococcus, PB19* and *MND1*. Thus, in Orchard A, WRR-N trees were found to be enriched for *Streptomyces* and *Bacillium* species when compared to WRR-infected trees.

In Orchard B, the comparison between WRR-S and WRR-N identified 20 significant genera (Figure 4D). WRR-S samples showed enrichment in *Rhodoblastus, Comamonas*, and *Rubrivivax* all in low abundance, while WRR-N samples were enriched in *Streptomyces, Pseudomonas, Pedomicrobium* and *Bacillus*. The comparison between WRR-S and WRR-AS revealed 29 significant genera (Figure 4E). In contrast to what was observed in Orchard A, WRR-S samples in Orchard B were enriched for *Bacillus* when compared to WRR-AS. WRR-AS was enriched in *Burkholderaceae, Cupriavidus*, and *Ramlibacter* although all in low relative abundance. When WRR-AS was compared to WRR-N, four significant genera were observed, with *Streptomyces* and *Pseudomonas* enriched in WRR-N (Figure 4F). As seen in Orchard A, WRR-N trees were found to be enriched for *Streptomyces* as compared to WRR-infected trees. In contrast to Orchard A, WRR-N in Orchard B was enriched for *Pseudomonas* when compared to both WRR-AS and WRR-S.

**Figure 5:**
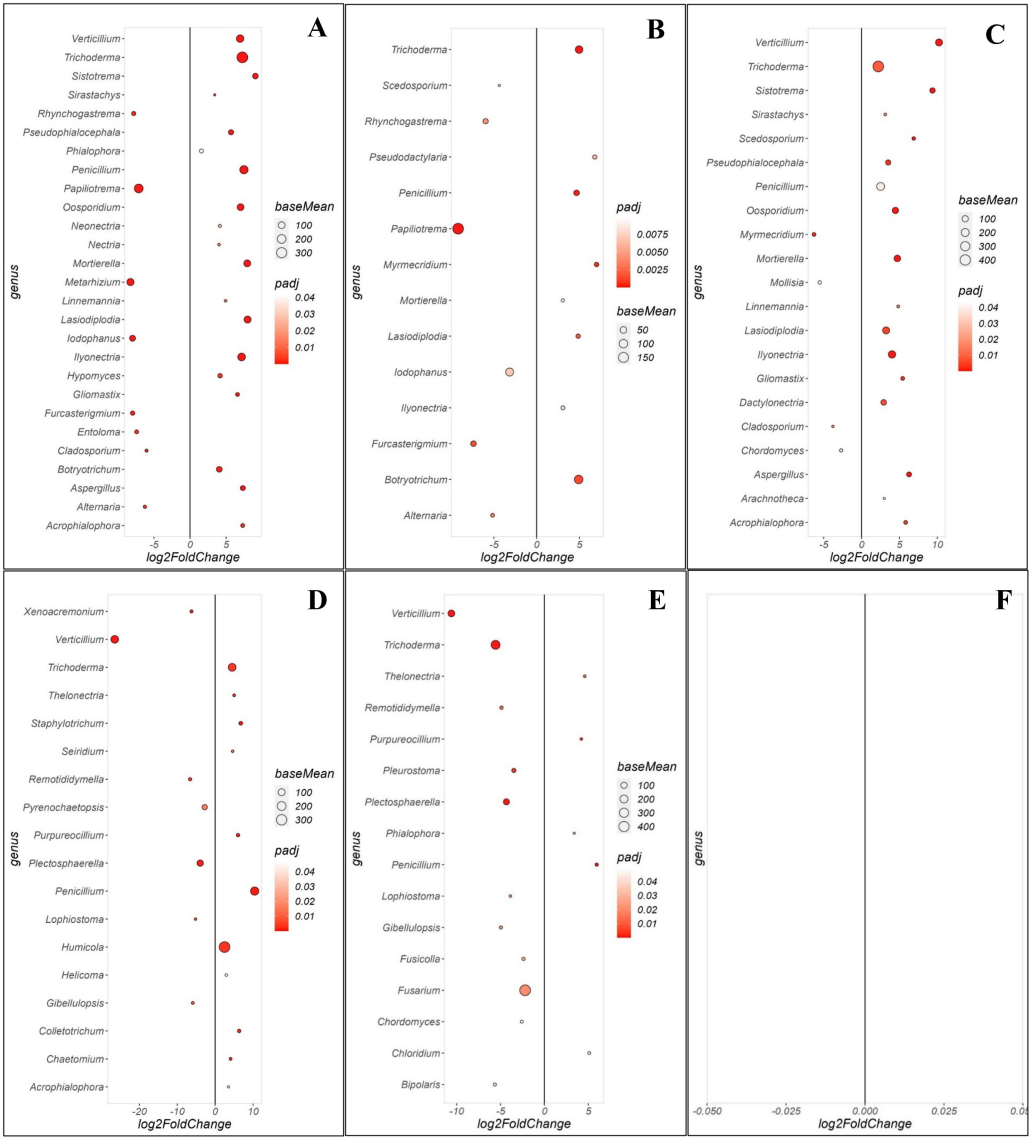
Comparison of specific fungal genera that were either enriched or depleted between different groups of soil samples collected in the rhizosphere at Orchards A and B. Comparison of rhizosphere soil samples between **(A)** WRR-S vs. WRR-N, **(B)** WRR-S vs. WRR-AS, **(C)** WRR-AS vs. WRR-N from Orchard A and **(D)** WRR-S vs. WRR-N, **(E)** WRR-S vs. WRR-AS, **(F)** WRR-AS vs. WRR-N from Orchard B. The group mentioned first was used as a reference when determining statistical significant differences; nj in the figure it will always have a positive log2fold change and thus be enriched in the reference group. The fold-change is shown on the X-axis and the genera are listed on the Y-axis. Each colored dot represents a genus population that is significantly more or less abundant (*p* ≤ 0.05). The size of the circle shows the average abundance, and the color intensity of the circle shows *padj* value (significance level). A larger circle indicates higher relative abundance in each population.

Fungal community comparisons revealed distinct enrichment patterns across the WRR-S, WRR-AS and WRR-N categories (Figure 5). When comparing WRR-S and WRR-N in Orchard A, 27 significant fungal genera were identified (Figure 5A). The WRR-S tree communities were enriched for *Papiliotrema* and *Metarhizium* in high relative abundance as well as *Alternaria* and *Cladosporium* in lower relative abundance. Nineteen genera were enriched in WRR-N including *Trichoderma, Penicillium* and *Ilyonectria* in high relative abundance while *Aspergillus, Lasiodiplodia, Verticillium* and *Mortierella* occured in lower relative abundance. The WRR-S vs. WRR-AS comparison identified 14 significant genera (Figure 5B). WRR-S showed enrichment in *Papiliotrema* and *Furcasterigmium* while WRR-AS samples were enriched in *Trichoderma, Botryotrichum Lasiodiplodia, Myrmecridium*, and *Penicillium*. The WRR-AS vs. WRR-N comparison revealed 21 enriched genera (Figure 5C), with WRR-N enriched in *Verticillium, Trichoderma, Mortierella, Lasiodiplodia, Ilyonectria Aspergillus*.

In Orchard B, the WRR-S vs. WRR-N comparison identified 18 significant fungal genera (Figure 5D). WRR-S was enriched with *Verticillium, Plectosphaerella* and *Pyrenochaetopsis*. In turn, WRR-N was enriched for *Humicola, Trichoderma* and *Penicillium* and these genera were all present in high relative abundance. The WRR-S vs. WRR-AS comparison identified 16 significant fungal genera (Figure 5E). WRR-S was enriched in *Fusarium, Verticillium*, and surprisingly *Trichoderma* which showed a high relative abundance in the WRR-S samples. WRR-AS was enriched in *Penicillium*, albeit in low relative abundance. However, the comparison between WRR-AS vs. WRR-N did not identify any significant fungal genera (Figure 5F). These results highlight the distinct fungal community shifts between soil categories in both orchards, but with some specific genera showing the same enrichment patterns across both orchards, such as *Trichoderma* and *Penicillium* in WRR-N as compared to WRR-S.

### Unique bacterial and fungal genera identified in the microbiomes of WRR-symptomatic, WRR-asymptomatic, and WRR-non infected avocado trees

We employed an UpSet plot to assess the number of unique genera in both the fungal and bacterial communities of the two orchards (Supplementary Figure 4), revealing notable differences between Orchard A and B. In Orchard A, there were 12 unique bacterial genera in WRR-S, 20 in WRR-AS and 25 in WRR-N (Supplementary Figure 4A, Supplementary Table 4). In Orchard B, there were 26 unique bacterial genera in WRR-S, 18 in WRR-AS and 21 in WRR-N (Supplementary Figure 4B, Supplementary Table 5). For unique fungal genera, Orchard A had 14 in WRR-S, 13 in WRR-AS and 22 in WRR-N (Supplementary Figure 4C, Supplementary Table 6). In Orchard B, 25 fungal genera were unique in WRR-S, 16 in WRR-AS and 21 in WRR-N (Supplementary Figure 4D, Supplementary Table 7).

Additionally, specific genera were unique, enriched and highly abundant within particular tree categories. *Chitinophaga* (Figure 4C, Supplementary Table 4); as well as *Gliomastix* and *Linnemannia* (Figure 5C, Supplementary Table 5) were found to be enriched, abundant and unique within the WRR-N samples from Orchard A. In Orchard B, *Acrophialophora* (Figure 5D, Supplementary Table 7) was enriched and unique to WRR-N. Similarly for genera within the WRR-S samples, *UTCFX1* (Figure 4E, Supplementary Table 5) and *Lophiostoma, Xenoacremonium, Gibellulopsis* and *Verticillium* (Figure 5D, Supplementary Table 7) were found to be enriched, abundant and unique in Orchard B. These results highlight significant variability in microbial community composition between orchards and categories, with key genera exhibiting potential ecological or functional relevance.

### Rhizosphere soil physicochemical properties of two avocado orchards

The physicochemical properties of rhizosphere soil from Orchards A and B were analyzed to evaluate the impact of *D. necatrix* on soil health (Table 3, Supplementary Tables 8, 9). Potassium (K) levels was significantly lower in Orchard A and significantly higher in Orchard B in WRR-S samples compared to WRR-AS or WRR-N. Low Phosphorus (P) levels were observed in the soil samples, with the lowest concentrations observed in WRR-S samples. In Orchard A, nitrogen (N) and magnesium (Mg) were significantly higher in healthy (WRR-N) samples than infected samples, while sodium (Na) and zinc (Zn) levels declined with increasing disease severity. Conversely, in Orchard B, pH, Na, and Zn were significantly higher in healthy samples (WRR-N) compared to WRR-S samples. The variability in soil properties between the two orchards highlights the potential influence of orchard-specific management practices, which appear to play a more critical role in shaping soil physicochemical characteristics than the direct effects of *D. necatrix*.

**Table 3:** Soil physicochemical properties of Orchard A and B.

**Orchard**	**Sample name**	**pH**	**N (mg/kg)**	**K (mg/kg)**	**P (mg/kg)**	**Mg (mg/kg)**	**Na (mg/kg)**	**Fe (mg/kg)**	**Zn (mg/kg)**
Orchard A	WRR-S	4.1 ± 0.11a	91.75 ± 6.5a	227.04 ± 22.4a	182.46 ± 8.6a	152.54 ± 10.2a	50.53 ± 5.8a	81.22 ± 5.83ac	50.53 ± 8.52a
	WRR-AS	4.4 ± 0.02ab	100.4 ± 1.7a	286.50 ± 20.2b	367.18 ± 5.0b	174.91 ± 4.7a	143.31 ± 4.81b	114.58 ± 11.62c	188.54 ± 7.68b
	WRR-N	5.0 ± 0.03bc	143.40 ± 2.9b	283.32 ± 13.3b	447.54 ± 14.2c	194.96 ± 10.3b	199.35 ± 9.6c	162.65 ± 7.5b	251.43 ± 3.6c
Orchard B	WRR-S	4.33 ± 0.05a	182.92 ± 4.1a	491.44 ± 9.8a	175.83 ± 13.5a	71.30 ± 5.9a	60.51 ± 7.1a	90.68 ± 3.1a	60.45 ± 1.6a
	WRR-AS	4.23 ± 0.09a	83.52 ± 1.7b	327.60 ± 14.5b	224.65 ± 14.1b	140.41 ± 11.5b	47.95 ± 6.8a	191.52 ± 3.10b	50.51 ± 6.8a
	WRR-N	5.38 ± 0.05b	103.08 ± 0.6b	385.24 ± 14.9b	384.93 ± 24.2c	230.79 ± 7.5c	187.9 ± 4.4b	195.08 ± 3.25b	235.2 ± 8.3b

Soil nutrients which were found to be significantly different in the rhizosphere soil between one or more of the tree categories [WRR symptomatic (WRR-S), WRR asymptomatic (WRR-AS), and healthy (WRR-N)] are shown. Values are the mean of the different soil physicochemical properties. The standard error is represented by ± values. For individual orchards, suffixes or different letters in the same vertical column indicate statistical significance at *p* < 0.05 using one way analysis of variance with sample name as the factor.

### The relationship between rhizosphere soil physicochemical properties and microbial community composition

**Figure 6:**
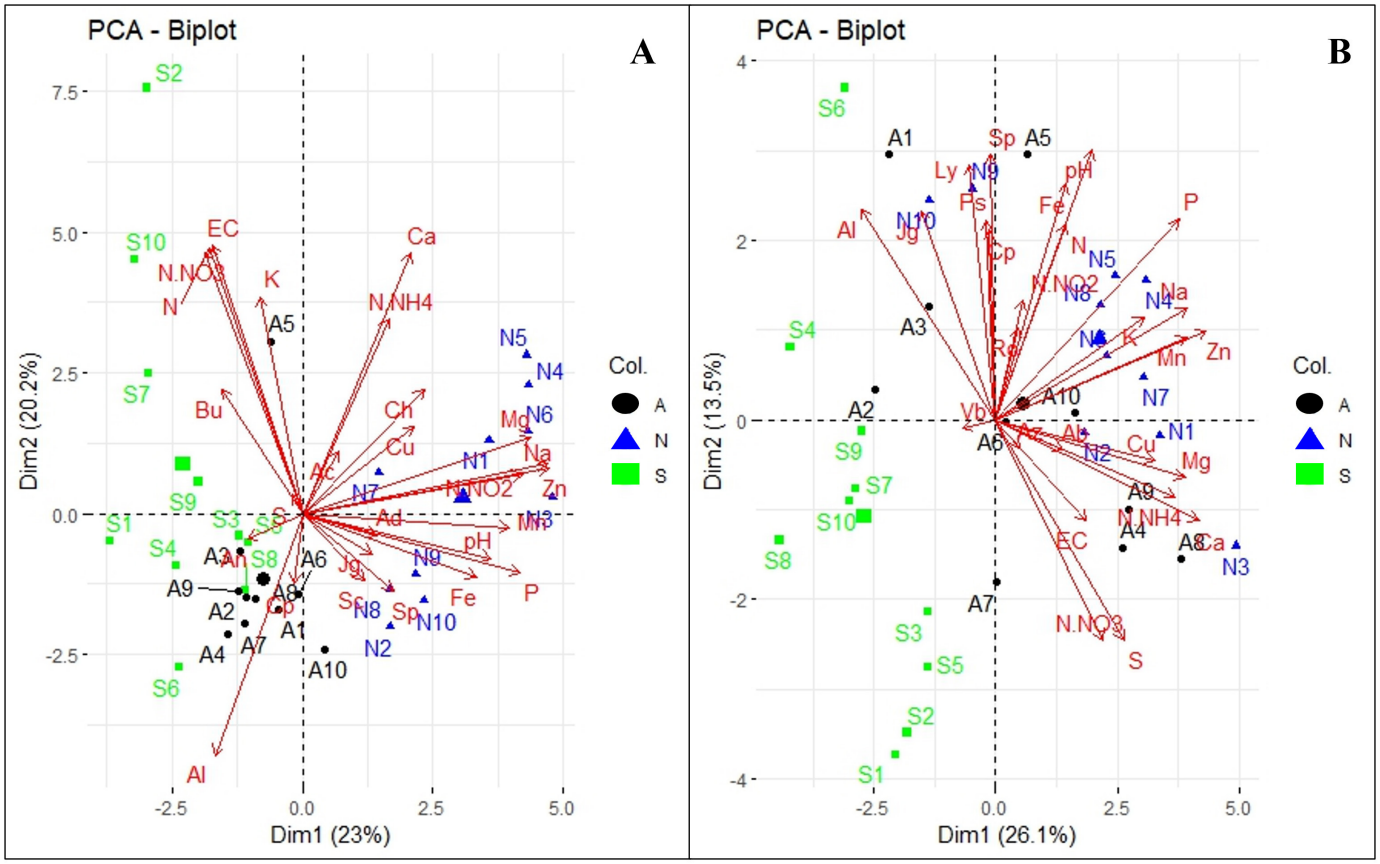
Principal Component Analysis (PCA) of abundant bacterial genera and soil physicochemical properties. **(A)** Orchard A and **(B)** Orchard B. The tree categories: WRR-S (S in green), WRR-AS (A in black) and WRR-N (N in blue). The top 0.6% bacterial genera: *Acidibacter* (Ac), *Acidothermus* (Ad), *Acinetobacter* (An), *Burkholderia-Caballeronia-Paraburkholderia* (Bu), *Chujaibacter* (Ch), *Cupriavidus* (Cp), *JG30-KF-AS9* (Jg)*, SC-I-84* (Sc), *Sphingomonas* (Sp)*, 11-24* (Ab), *Azohydromonas* (Az), *Lysobacter* (Ly), *Pseudomonas* (Ps), *Rokubacteriales* (Ro) and *Vicinamibacteraceae* (Vb). Soil physicochemical properties: exchangeable cations (EC), potential of hydrogen (pH), Nitrogen dioxide (N-NO_2_), Nitrate Nitrogen (N-NO_3_), Ammonium nitrate (N-NH_4_), Nitrogen (N), Potassium (K), Phosphorus (P), Magnesium (Mg), Calcium (Ca), Sodium (Na), Sulphur (S), Aluminium (Al), Copper (Cu), Manganese (Mn), Iron (Fe), Zinc (Zn). The arrow lengths in the plot represent the association strength between the soil physicochemical properties and the bacterial genera (the longer the arrows, the stronger the association). The perpendicular distance between microbes and soil physicochemical properties axes reflects their correlation (the smaller the distance, the stronger the association).

To investigate the association between soil physicohemical properties and microbial communities, dominant fungal and bacterial genera (>0.6%) were selected for principal component analysis (PCA). The analysis revealed distinct patterns in microbial associations and soil properties across Orchards A and B (Figure 6), with variations contributing to the observed clustering. In Orchard A, the analysis showed that dimension 1 and 2 explained 23 and 20.2% of the total variance (Figure 6A). WRR-AS samples (in black) clustered negatively along Dim1, with correlations with *Acinetobacter* (An) and *Cupriavidus* (Cp), as well as Al. WRR-S samples (in green) clustered negatively on Dim1 but varied across Dim2, correlating positively with N-NO_3_, N, K, EC, and the *Burkholderia-Caballeronia-Paraburkholderia* complex (Bu). WRR-N samples (in blue) clustered positively on Dim1, and associated with pH, Mg, Na, Zn, N-NO_2_, Mn, available P, Cu, Fe, and bacterial genera like *Sphingomonas* (Sp) and *Chujaibacter* (Ch).

In Orchard B, the PCA of thee bacterial genera and soil propeerties showed that the two dimensions combined contributed 39.6% of the variability observed in the dataset (Figure 6B). WRR-S samples (in green) clustered primarily on the negative side of Dim1 and Dim2, indicating a negative association with soil properties and bacterial genera. In contrast, WRR-N (in blue) and WRR-AS samples (in black) clustered positively along Dim1, demonstrating strong associations with soil factors such as pH, Fe, N, available P, exchangeable K, Mg, Ca, Na, Zn, Mn, Cu, S, N-NH_4_, N-NO_3_, and EC. These samples also correlated with bacterial genera such as *Azohydromonas* (Az), and members of the *Rokubacteriales* (Ro) order.

**Figure 7:**
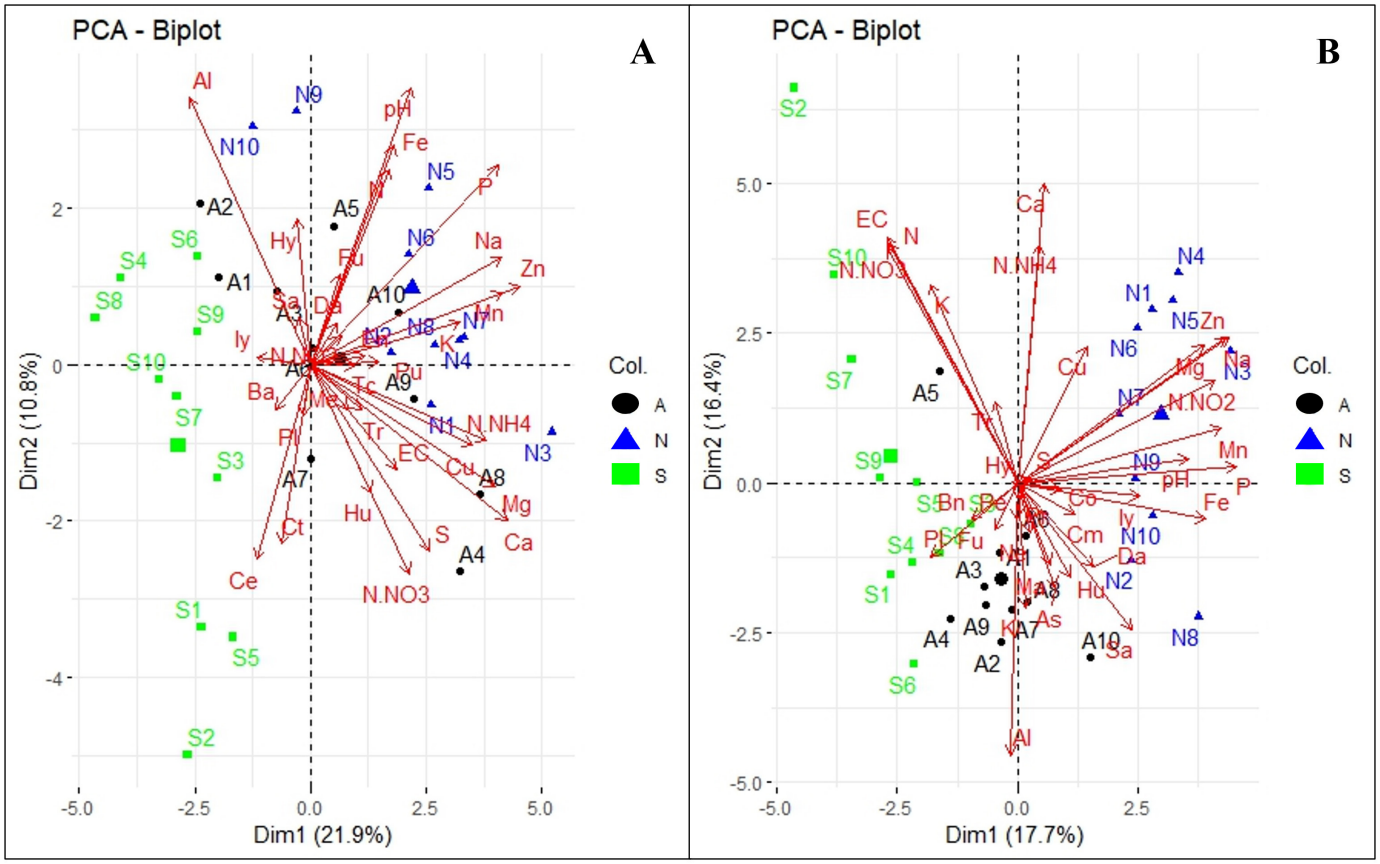
Principal Component Analysis (PCA) of abundant fungal genera and soil physicochemical properties. **(A)** Orchard A and **(B)** Orchard B. The tree categories: WRR-S (S in green), WRR-AS (A in black) and WRR-N (N in blue). The top 0.6% fungal genera: *Bartalinia* (Ba) *Cephalotrichum*, (Ct), *Cercophora* (Ce), *Dactylonectria*, (Da), *Enterocarpus* (En), *Fusarium* (Fu), *Humicola* (Hu), *Hyaloscypha* (Hy), *Ilyonectria* (Iy)*, Metarhizium* (Me), *Plectosphaerella* (PI), *Pseudallescheria* (Pu), *Saitozyma* (Sa), *Trichocladium* (Tc), *Trichoderma* (Tr), *Aspergillus* (As), *Barnettozyma* (Bn), *Chloridium* (Cm), *Cosmospora* (Co), *Kendrickiella* (KI), *Mariannaea* (Ma), *Neopyrenochaeta* (Ne), *Paracremonium* (Pr), and *Penicillium* (Pe). Soil physicochemical properties: exchangeable cations (EC), potential of hydrogen (pH), Nitrogen dioxide (N-NO_2_), Nitrate Nitrogen (N-NO_3_), Ammonium nitrate (N-NH_4_), Nitrogen (N), Potassium (K), Phosphorus (P), Magnesium (Mg), Calcium (Ca), Sodium (Na), Sulphur (S), Aluminium (Al), Copper (Cu), Manganese (Mn), Iron (Fe), Zinc (Zn). The arrow lengths in the plot represent the association strength between the soil physicochemical properties and the fungal genera (the longer the arrows, the stronger the association). The perpendicular distance between microbes and soil physicochemical properties axes reflects their association (the smaller the distance, the stronger the association).

For fungal genera in Orchard A, Dim1 and Dim2 explained 10.8 and 21.9% of the total variance, respectively (Figure 7A). WRR-S samples (in green) were associated with genera such as *Ilyonectria* (Iy), *Bartalinia* (Ba), *Cephalotrichum* (Ct), and *Cercophora* (Ce). In contrast, WRR-N (in blue) and WRR-AS samples (in black) clustered positively along Dim2, correlating with fungal genera including *Fusarium* (Fu), *Enterocarpus* (En), *Trichocladium* (Tc), *Dactylonectria* (Da), *Trichoderma* (Tr), *Pseudallescheria* (Pu), *Metarhizium* (Me) and *Humicola* (Hu), as well as soil properties like pH, P, N, Fe, Na, Mn, Zn, and K.

Orchard B exhbited similar fungal profiles, with dimensions accounting for 16.4% and 17.7% of the total variance, respectively (Figure 7B). WRR-S samples (in green) clustered negatively along Dim1 and were associated with N-NO_3_, N, K, and EC, and fungal genera such as *Plectosphaerella* (PI) and *Fusarium* (Fu). WRR-AS samples (in black) occupied a central position and were linked to *Penicillium* (Pe), *Fusarium* (Fu), and *Kendrickiella* (KI), as well as Al. WRR-N samples (in blue) were associated with *Ilyonectria* (Iy) as well as pH, Fe, P, Mn, N-NO_2_, Na and Zn.

Overall, the PCA results demonstrated distinct microbial and soil properties across disease categories and orchards. While there were similarities in the fungal and bacterial profiles of the two orchards, differences in clustering patterns were observed. These findings might underscore the complexity of soil-microbial interactions and their relationship to environmental conditions and orchard management.

### *In vitro* antagonistic effect of fungi and bacteria against *D. necatrix*

**Figure 8:**
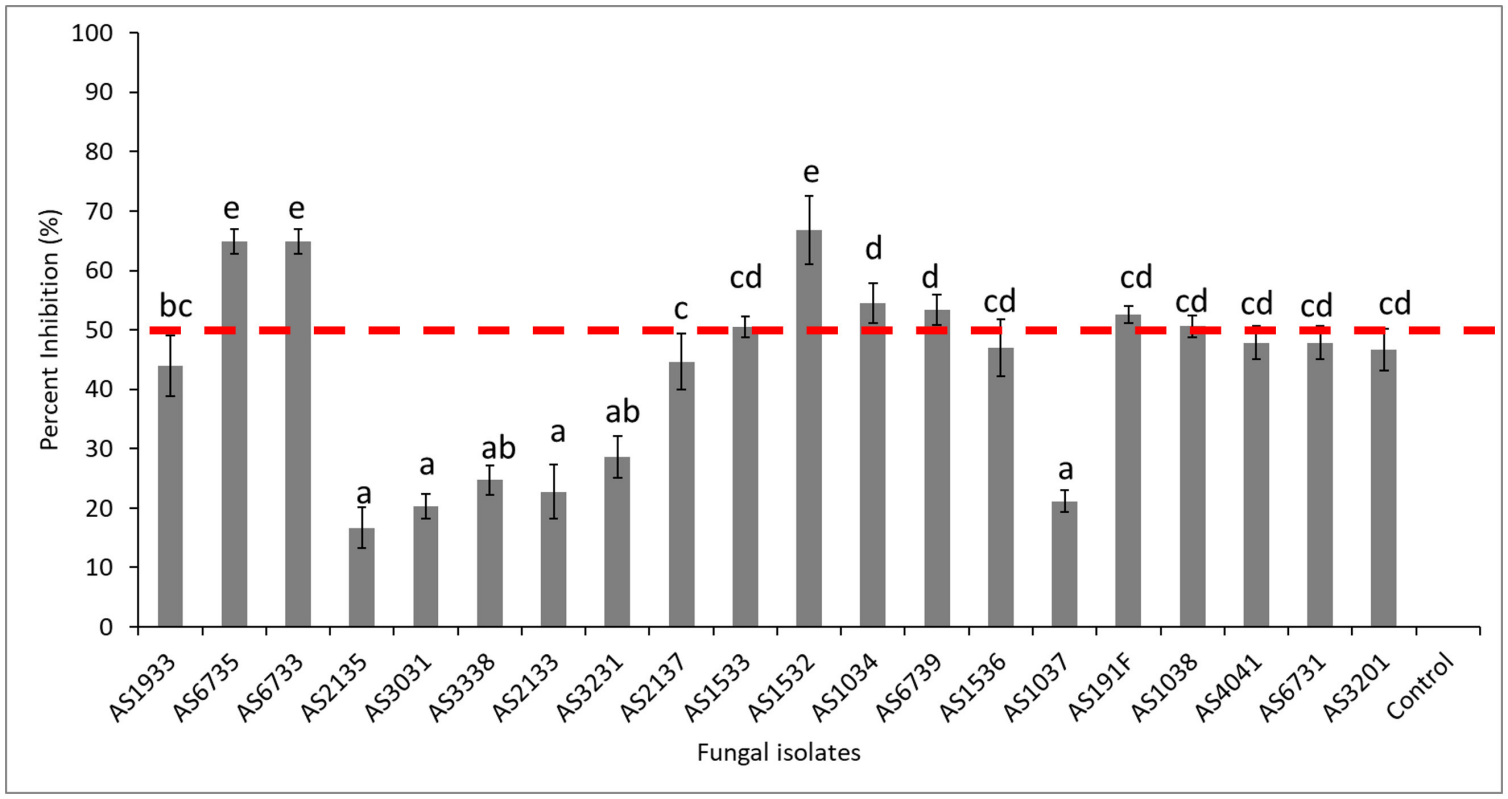
Percent inhibition (%) of selected fungal isolates showing antagonistic activity against *Dematophora necatrix*. AS1034 (*Mortierella alpina*), AS1037 (*Penicillium chrysogenum*), AS1038 (*Penicillium citreonigrum*), AS1532 (*Fusarium solani*), AS1533 (*Fusarium solani*), AS1536 (*Aspergillus flavus*), AS191F (*Linnemannia elongate*), AS1933 (*Cladosporium halotolerans*), AS2133 (*Dactylonectria novozelandica*), AS2135 (*Neopyrenochaeta acicola*), AS2137 (*Penicillium expansum*), AS3031 (*Subramaniula cuniculorum*), AS3201 (*Geomyces asperulatus*), AS3231 (*Purpureocillium lilacinum*), AS3338 (*Gloeotinia temulenta*), AS4041 (*Penicillium buchwaldii*), AS6731 (*Talaromyces variabilis*), AS6733 (*Fusarium solani*), AS6735 (*Trichoderma longibrachiatum*), AS6739 (*Podila minutissima*), and Control (*Dematophora necatrix* only). The mean inhibition rates indicated by the same letter are not significantly different according to Tukey's test (*p* ≤ 0.05).

**Figure 9:**
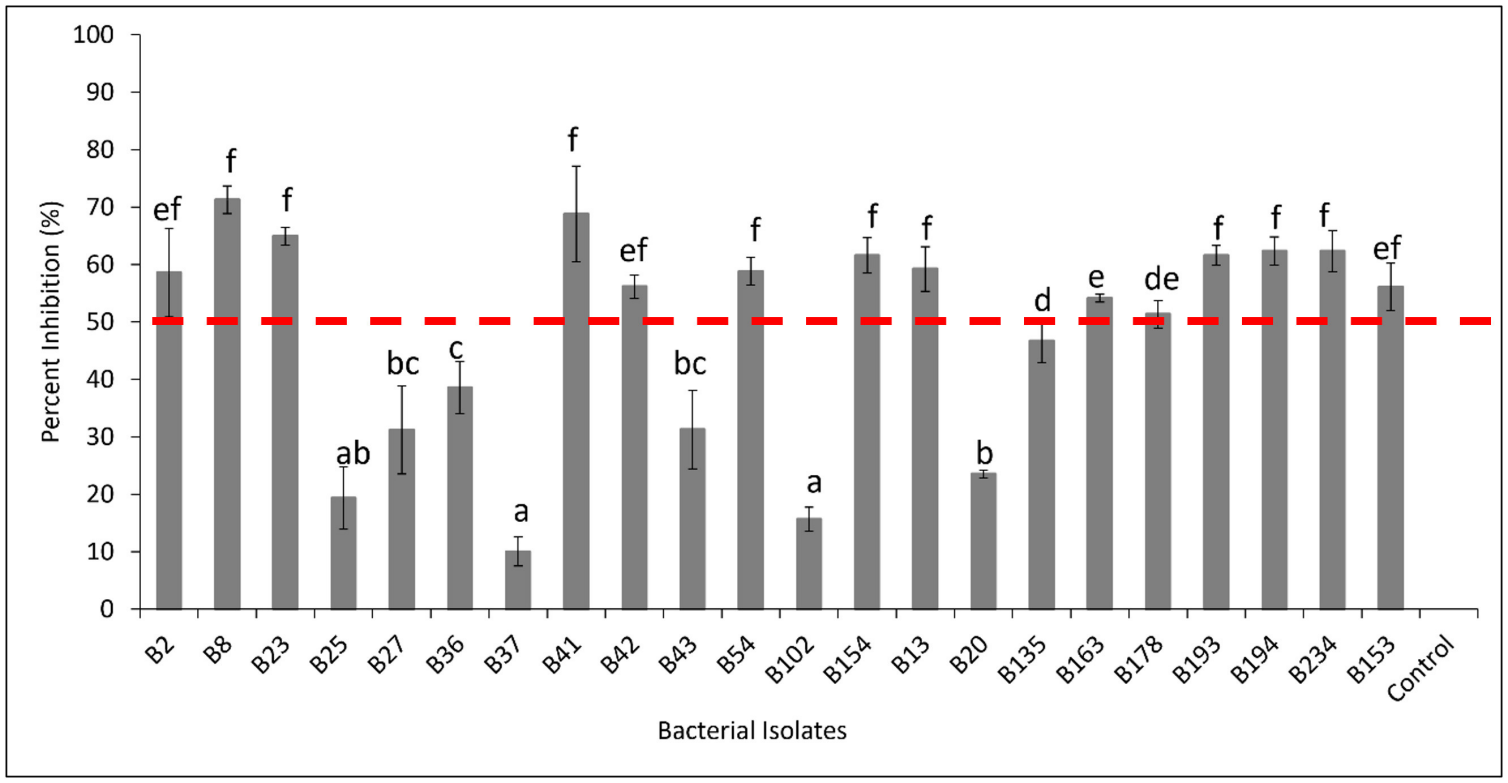
Percent inhibition (%) of selected bacterial isolates showing antagonistic activity against *Dematophora necatrix*. B102 (*Serratia odorifera*), B13 (*Stremptomyces* sp.), B135 (*Pseudomonas baetica*), B153 (*Pseudomonas koreensis*), B154 (*Pseudomonas koreensis*), B163 (*Pseudomonas koreensis*), B178 (*Pseudomonas fluorenscens*), B193 (*Psedomonas koreensis*), B194 (*Pseudomonas fluorescens*), B2 (*Bacillus subtilis*), B20 (*Serratia odorifera*), B23 (*Bacillus subtilis*), B234 (*Pseudomonas* sp.), B25 (*Microbactrium testaceum*), B27 (*Microbacterium* sp.), B36 (*Microbactrium testaceum*), B37 (*Microbactrium testaceum*), B41 (*Bacillus tequilensis*), B42 (*Bacillus subtilis*), B43 (*Microbacterium trichothecenolyticum*), B54 (*Pseudomonas* sp.), B8 (*Bacillus subtilis*) and Control (*Dematophora necatrix* only). The mean inhibition rates indicated by the same letter are not significantly different according to Tukey's test (*p* ≤ 0.05).

A total of 20 fungal and 22 bacterial isolates were isolated from healthy soil and assessed for their ability to inhibit *D. necatrix* growth (Supplementary Tables 10, 11, respectively). Among the fungal isolates, eight demonstrated significant inhibitory activity, reducing the mycelial growth of *D. necatrix* by more than 50%. These isolates included *Trichoderma longibrachiatum* (AS6735), *Fusarium solani* (AS6733, AS1533, and AS1532), *Mortierella alpina* (AS1034), *Podila minutissima* (AS6739), *Linnemannia elongate* (AS191F) and *Penicillium citreonigrum* (AS1038) (Figure 8). The remaining 12 fungal isolates showed less than 50% inhibition, indicating limited effectiveness against *D. necatrix* (Figure 9).

Among the bacterial isolates, 14 exhbited strong inhibitory effects, reducing *D. necatrix* growth with more than 50%. These isolates included *Bacillus subtilis* (B2, B8, B23 and B42), *Bacillus tequilensis* (B41), *Pseudomonas* sp. (B54 and B234), *Pseudomonas koreensis* (B154, B163, B193, B153), *Pseudomonas fluorenscens* (B178 and B194), and *Streptomyces* sp. (B13) (Figure 9). These results highlight the potential of several fungal and bacterial isolates as biocontrol agents against *D. necatrix*, with particular promise shown by members of the *Trichoderma, Fusarium, Pseudomonas*, and *Bacillus* genera. Further studies are needed to explore their mechanisms of action and potential application in soil disease management.

## Discussion

This study investigated the bacterial and fungal communities in the rhizosphere of WRR- asymptomatic (WRR-AS), symptomatic (WRR-S), and non-infected (WRR-N) avocado trees, along with soil health parameters, to assess the impact of *D. necatrix* on microbial composition and diversity. The presence of *D. necatrix* in both orchards confirmed earlier reports by van den Berg et al. (2018) for South African avocado orchards. Consistent with global findings, *P. cinnamomi* was also detected in all samples of this study (Duvenhage et al., 1991; Pérez-Jiménez, 2008; Engelbrecht and van den Berg, 2013; Ramírez-Gil et al., 2017). Interestingly, *D. necatrix* did not significantly alter the overall diversity of the avocado rhizosphere microbiome.

The genera *Fusarium* and *Sphingomonas* were the only abundant genera across WRR-S, WRR-AS, and WRR-N soil samples in both orchards. WRR-N samples showed stronger associations with key soil physicochemical properties, and fungal and bacterial microbial composition, indicating healthy soil in areas where *D. necatrix* was absent. Beneficial microorganisms such as *Trichoderma, Streptomyces* and *Bacillus* were more enriched in WRR-N samples in both orchards, while *Pseudomonas* was only prevalent in healthy soil in Orchard B (Iacomino et al., 2022; Singh et al., 2022; Prigigallo et al., 2023). Notably, isolates of *Bacillus, Pseudomonas, Trichoderma* and *Penicillium* recovered from healthy soil in this study demonstrated significant inhibition of *D. necatrix* growth in dual-culture assays, highlighting their potential use as biocontrol agents. A previous study demonstrated the inoculation of the *Morchella importuna* mycosphere with *Pseudomonas chlororaphis* led to reduction of *Paecilomyces penicillatus*, indicating a restoration of a healthy soil microbiome and enhanced biocontrol properties (Yu et al., 2025). These findings provide valuable insights into the interaction between *D. necatrix* and the avocado rhizosphere, offering potential avenues for effective disease management.

### The bacterial and fungal diversity of the avocado rhizosphere is not altered by *D. necatrix*

Contrary to our hypothesis, *D. necatrix* did not significantly alter the microbiome in the two avocado orchards studied, this is consistent with previous findings for *P. cinnamomi*, which similarly did also not affect species richness or diversity in the avocado rhizosphere (Solís-García et al., 2021). The lack of *D. necatrix* on diversity metrics could be due to various factors, such as the buffering effect of *P. cinnamomi*, and the plant defense response. The gap in knowledge may be addressed using in depth root exudates analysis which can clarify the interactions between pathogens and their effect on the rhizosphere microbiome. A similar lack of impact on the diversity of the rhizosphere fungal communities was reported for kiwifruit vine decline syndrome-affected kiwifruit plants when compared to healthy plants (Guaschino et al., 2023).

### Abundance of bacterial genera of two avocado orchards

In Orchard A, the shared most abundant bacterial genera across WRR-S, WRR-AS, and WRR-N included *Sphingomonas, SC-I-84, Rokubacteriales*, and *Lysobacter*. The presence of *Sphingomonas*, which has previously been shown to be associated with plant stress (Asaf et al., 2020), suggests stress due to the presence of *P. cinnamomi* and *D. necatrix*. Additionally, *Lysobacter*, a known biological control agent (BCA) against *Fusarium oxysporum, Colletotrichum gloeosporiodes*, and *Rhizoctonia* spp., was similarly identified in the rhizosphere of avocado trees exhibiting Fusarium dieback (Bejarano-Bolívar et al., 2021). Interestingly, *Pseudomonas* was abundant only in WRR-S samples in Orchard A. This may reflect plant responses to pathogen stress through the release of root exudates as plants under stress may recruit beneficial microbes to enhance defenses (Rolfe et al., 2019; Qu et al., 2020). In orchard B however, *Pseudomonas* was associated with WRR-N samples, which may be related to local soil properties, host age or genotype, rather than to the presence/absence of *D. necatrix*. The presence of *P. cinnamomi* in avocado orchards was previously shown to increase the abundance of Pseudomonades and Burkholderiales in the rhizosphere, while reducing the abundance of *Actinobacteria, Bacillus*, and Rhizobiales (Solís-García et al., 2021).

In Orchard B, *Sphingomonas, SC-I-84*, and *Acidothermus* were the most abundant shared genera across all samples. *Acidothermus*, known for increasing soil nutrient content by breaking down cellulose (Lin et al., 2022) was less abundant in WRR-S and WRR-AS compared to healthy soil (WRR-N), possibly reflecting reduced biomass utilization in diseased trees (Ren et al., 2021). The recruitment of *Sphingomonas*, observed in high abundance in this study, may have been triggered by root exudates in response to *P. cinnamomi* (present in all samples) and *D. necatrix*, as similar mechanisms have been reported where root exudates from cucumber infected with *Fusarium oxysporum* f.sp. *cucumerinum* attract beneficial microbes to counter pathogen invasion (Wen et al., 2023). These findings highlight the potential role of rhizosphere bacteria in mitigating plant stress and maintaining soil health.

### The enrichment of specific bacterial genera between WRR-S, WRR-AS and WRR-N categories of avocado trees

Enrichment refers to the increased prevalence or activity of specific microorganisms driven by environmental conditions or pathogen presence. *Pseudomonas*, known for its ability to evade or suppress plant defenses and colonize the rhizosphere (Liu et al., 2018; Mendes et al., 2011; Yu et al., 2019), was abundant in WRR-S samples consistent with findings by Solís-García et al. (2021). The authors reported the presence of Pseudomonadales, Burkholderiales, Beta- and Gamma-Proteobacteria in the rhizosphere of root rot symptomatic avocado trees. These genera are fast-growing colonizers supported by carbon rich root exudates released under stress and by rotting roots (Eilers et al., 2010; Trivedi et al., 2012).

In Orchard B, WRR-N samples were enriched with *Streptomyces* (Actinobacteria), *Pseudomonas*, and *Bacillus* (Firmicutes). Previous studies linked Acidobacteria, Actinobacteria and Firmicutes with asymptomatic avocado trees infected with *P. cinnamomi* (Solís-García et al., 2021), supporting our results. The presence of *Streptomyces* in WRR-N may offer disease-suppressive potential, as a reduction in *Streptomyces* sp. has been linked to the lack of disease suppression in strawberry plants (Kim et al., 2019). *Bacillus* sp., typically associated with healthy samples in this study, are known to suppress *Phytophtora* root rot (PRR) in avocado (Guevara-Avendaño et al., 2018). These findings underscore the potential of beneficial microbes like *Streptomyces* and *Bacillus* in managing WRR and other opportunistic pathogens in avocado rhizospheres.

### Abundance and enrichment of fungal genera in two avocado orchards

*Ascomycetes* and *Basidiomycetes* were the dominant fungi in both avocado orchards. In Orchard A, *Fusarium* and *Cercophora* were the most prevalent genera across all categories. The presence of the pathogens *Ilyonectria* and *Dactylonectria* in WRR-N samples was not unexpected as these samples were positive for *P. cinnamomi*, and these genere were previously reported in the pathobiome of avocado trees infected with *P. cinnamomi* (Solís-García et al., 2021).

Orchard B exhibited dominance of *Fusarium, Saitozyma*, and *Hyaloscypha* across all three categories. Similarly to Orchard A, WRR-N was characterized by *Trichoderma, Penicillium* and the two *Nectriaceae* pathogens. WRR-S featured *Trichoderma* and *Paracremonium*. These findings align with studies on the impact of *Phytophthora* on rhizosphere microbiomes (Solís-García et al., 2021; Reverchon et al., 2023). *Penicillium* and *Trichoderma* were detected in WRR-N trees, in agreement with Handique et al. (2024), who found the microbes more abundant in uninfected Khasi Mandarin as compared to *Phytophthora* infected-Khasi Mandarin citrus trees.

Previous research has indicated that *P. cinnamomi* can alter the microbial structure and composition of the avocado rhizospheres and is often associated with other pathogens that contribute to root rot (Yang et al., 2001; Shu et al., 2019; Solís-García et al., 2021). This may explain the co-existence of *P. cinnamomi, D. necatrix* and opportunistic genera such as *Fusarium* in our study; *Fusarium* spp. were prevalent in both orchards, particularly in WRR-S samples. Our findings align with studies on *Phytophthora*-infected soil in the rhizosphere of citrus, where *Fusarium* thrives in pathogen-stressed soils (Handique et al., 2024). Infected citrus plants also exhibited high abundance of *Aspergillus, Saitozyma* and *Colletotrichum*, while non-infected plants were dominated by *Trichoderma, Penicillium, Mortierella* and *Saitozyma* (Handique et al., 2024). While the Fusarium genus contains several notable pathogenic species; such as *Fusarium solani, F. oxysporum, F. equisetti* and *F. chlamydosporum* (Burgess et al., 1994; Chimbekujwo, 2000; Crous et al., 2021); most isolates that are abundant in the soil, frequently associate with plant roots as saprophytes (Nelson et al., 1994). Some *Fusarium* spp. may even act as endophytes of avocado and have even been reported to have anti-oomycete activity and plant growth promoting properties (Nieves-Campos et al., 2024).

The presence of *Ilyonectria* (previously classified under *Cylindrocarpon*) in both avocado orchards is concerning, as it is linked to root rot, stem lesions, damping off, branch and crown cankers, fruit disease, reduced yield, and increased susceptibility to other pathogens (Chaverri et al., 2011). *Cylindrocarpon destructans, Cylindrocladium parvum* and less often *Cylindrocladium scoparium* were isolated in South African avocado orchards in the 1970s (Darvas, 1978), while *Cylindrocarpon* sp. was isolated in Spain in the 1980s from severly affected avocado trees (López-Herrera and Melero-Vara, 1992). These studies have suggested that these fungi exacerbate vulnerability to *D. necatrix* and *P. cinnamomi*. Similarly, *Calonectria, Cylindrocladiella* and *Neonectria* have been reported from diseased avocado roots in Australia (Dann et al., 2012).

The co-occurence of *D. necatrix* and *P. cinnamomi, Ilyonectria* and opportunistic pathogens like *Fusarium*, suggests a disrupted microbial community structure that impacts avocado tree health. The South African avocado industry is ill-equipped to manage such fungal threats, especially as climate change increases pathogen pressures. Similar challenges have already been observed with *Ilyonectria* in avocado orchards in Spain and Australia (López-Herrera and Melero-Vara, 1992; Dann et al., 2012). Without effective intervention, pathogens like *Fusarium* and *Ilyonectria* could become increasingly dominant, posing a severe threat to avocado cultivation.

As expected, *Trichoderma* was abundant and enriched in WRR-N soil in the two orchards, suggesting its potential as an indicator of tree health. Known for its biocontrol properties, *Trichoderma* has been shown to inhibit *D. necatrix* effectively in laboratory and greenhouse studies (Freeman et al., 1986; Ruano-Rosa and López-Herrera, 2009; Ruano-Rosa et al., 2010). Its presence highlights the importance of beneficial fungi in maintaining soil health and suppressing diseases.

### Unique bacterial and fungal genera between WRR-S, WRR-AS and WRR-N categories of avocado trees

The presence of unique genera offers opportunities to discover beneficial microbes for pathogen management and to preserve microbial diversity. The current study identified distinct fungal and bacterial genera associated with WRR-S, WRR-AS, and WRR-N categories, with some genera uniquely enriched in specific groups. In Orchard A, *Chitinophaga* was enriched and unique to WRR-N. This genus, belonging to the phylum Bacteroidetes, is involved in plant biomass degradation (Funnicelli et al., 2021). Previous studies showed that *Chitinophaga flava* isolated from the rhizosphere soil of tomato plants, demonstrated high levels of antifungal activity against gray mold disease caused by *Botrytis cinerea*, exhibiting potential biocontrol activity (Kim et al., 2023). Also unique to WRR-N in Orchard A were *Gliomastix* and *Linnemannia*, with species like *Linnemannia elongate* playing a role in chitin-based plant growth promotion (De Tender et al., 2024). In Orchard B, *Bartalinia* was highly abundant and unique to WRR-AS. Very little is known about this wood-decomposing genus in South Africa (Marincowitz et al., 2010).

No unique and abundant fungi were identified in orchard A. In Orchard B, *Colletotrichum, Lophiostoma, Xenoacremonium, Gibellulopsis, Bipolaris* and *Verticillium* were enriched and unique to WRR-S samples. Many of these genera are known plant pathogens causing significant losses (Barbara and Clewes, 2003; De Silva et al., 2017; Al-Sadi, 2021; Amani et al., 2023). *Colletotrichum* species are primarily considered foliar and fruit pathogens, causing anthracnose diseases in a wide range of crops such as red rot of sugarcane, crown rot of strawberry, and banana (Lenné, 2002). However, some species, such as *Colletotrichum graminicola* in maize, have been reported as soilborne pathogens, persisting in soil as saprophytes, surviving on plant debris and infecting plants through root-associated interactions (Sukno et al., 2008). *Verticillium dahlia*, for example, secretes effectors that manipulate bacterial microbiomes promoting infection (Snelders et al., 2022). The presence of *Verticillium* in WRR-S avocado plants may contribute to increased susceptibility and disease progression. A similar phenomenon has been reported with *D. necatrix* where microbial interactions exacerbated disease severity in cotton (Chavarro-Carrero et al., 2024). The findings suggest that WRR infected trees attract opportunistic pathogens, reducing resilience against infections, as previously observed in avocado orchards affected by PRR (Solís-García et al., 2021). WRR significantly alters the rhizosphere microbiome, enriching unique genera in both infected and non-infected trees, which may influence disease dynamics and plant health.

### The association of microbial communities with soil physicochemical analysis

The study demonstrated a strong association between fungal and bacterial microbial community structure and rhizosphere soil properties in WRR-N samples compared to WRR-S and WRR-AS samples. For example, soil pH and Fe were strongly related with several bacteria and fungi, emphasizing the key role pH plays in shaping microbial diversity and driving processes like nitrification and denitrification (Hayatsu et al., 2021). This distinction may be attributed to enhanced microbial activity, which facilitates nutrient cycling and organic matter decomposition, ultimately improving soil fertility and plant growth (Condron et al., 2010). The observed association highlight the intricate relationship between soil physicochemical properties and microbial ecology, reinforcing the importance of maintaining healthy soils for sustainable productivity. Finally, WRR-N soils clustered on the opposite side of the PCA to WRR-AS and WRR-S soils, together with several soil properties; this may indicate patterns likely to support a decreased incidence of WRR.

### Screening and determination of antagonistic effects of culturable bacteria and fungi

This study identified bacterial and fungal microorganisms from healthy soil and demonstated antagonistic activity against *D. necatrix*. The characterization of antagonistic activity and activities associated with plant growth has previously enabled the identification of BCAs against *D. necatrix* (Pliego et al., 2007; Ruano-Rosa et al., 2010). Dual-culture assays showed that bacterial genera such as *Pseudomonas, Bacillus* and *Streptomyces* effectively inhibited mycelial growth of *D. necatrix*, consistent with their role in disease suppression and plant growth promotion. For example, *Pseudomonas chlororaphis* PCL1606 exhibits antifungal activity against *D. necatrix* by competing for space and root exudate nutrients (Calderón et al., 2014). Additionally in support of our findings, *B. subtilis* strains isolated from healthy avocado rhizospheres have demonstrated antifungal activity against *D. necatrix* in both *in vitro* and *in vivo* studies, directly attacking soil pathogens and stimulating plant defenses (Cazorla et al., 2007). Similarly, rhizosphere isolates of *Bacillus* have been shown to reduce mycelial growth of *Fusarium euwallaceae*, a causal agent of Fusarium dieback in avocado (Guevara-Avendaño et al., 2019). These findings suggest the potential of bacteria with antifungal properties to mitigate *D. necatrix* in avocado orchards.

Fungal genera such as *Trichoderma, Penicillium*, and *Mortierella* also inhibited *D. necatrix* in dual-culture assays. *Trichoderma* is well known for promoting plant growth and suppressing soil-borne diseases, with previous studies demonstrating its efficacy against *D. necatrix in vitro* and in greenhouse trials (Freeman et al., 1986; Ruano-Rosa et al., 2010). Additionally, *Fusarium* and *Aspergillus* species also showed antagonistic activity. This suggests that there is intense competition within the avocado rhizosphere, where *D. necatrix* must contend with other fungal pathogens to establish infection. Future studies should prioritize field trials with promising fungal and bacterial isolates to develop effective biocontrol strategies for avocado orchards.

## Conclusion

This study provides valuable insights into the microbial dynamics of avocado rhizospheres infected with *D. necatrix* and highlights potential biocontrol organisms. While *D. necatrix* did not significantly alter overall microbial diversity, its presence, alongside *P. cinnamomi*, influenced the enrichment of specific bacterial and fungal genera, shaping the rhizosphere composition. Beneficial genera such as *Trichoderma, Bacillus, Streptomyces*, and *Pseudomonas* were associated with WRR-N soils, reflecting a potential to develop a more resilient rhizosphere microbiome and enhanced disease suppression potential compared to WRR-S and WRR-AS soils. Future research should focus on pathogenicity trials in the greenhouse and field trials to validate the efficacy of identified microbial biocontrol agents under orchard conditions and further explore the mechanisms underlying microbial interactions. Additionally, metagenomic, metatranscriptomic, and, metabolomic approaches could provide comprehensive insights into the functional roles of rhizosphere microbiota, enabling tailored interventions to enhance avocado tree health and resilience.

## Data Availability

The datasets presented in this study can be found in online repositories. The names of the repository/repositories and accession number(s) can be found in the article/Supplementary material.
