# Supplementary material for: Avocado rhizosphere community profiling: white root rot and its impact on microbial composition
Source: Front Microbiol. 2025 May 23;16:1583797. doi: 10.3389/fmicb.2025.1583797 (PMC12141342; doi:10.3389/fmicb.2025.1583797)
Supplement: Supplementary file 1 [file Data_Sheet_1.docx]

**Avocado Rhizosphere Community Profiling: White Root Rot and its Impact on Microbial Composition**

**Phinda Magagula^1,2^, Velushka Swart^2,3^, Arista Fourie^4^, Alicia Vermeulen^2^, Johannes Harold Nelson^2,3^,** **Zelda van Rooyen^5^, Noëlani van den Berg^2,3^***

^1^Department of Plant and Soil Sciences, University of Pretoria, Pretoria, South Africa

^2^Hans Merensky Chair in Avocado Research, Forestry and Agricultural Biotechnology Institute (FABI), University of Pretoria, Pretoria, South Africa

^3^Department of Biochemistry, Genetics and Microbiology, University of Pretoria, Pretoria, South Africa

^4^Theoretical Biology and Bioinformatics, Department of Biology, Science for Life, Utrecht University, Utrecht, The Netherlands

^5^Westfalia iTeam, Westfalia Fruit, Tzaneen, South Africa

**Submitted Journal:**

Frontiers in Microbiology

***Correspondence:**

Noëlani van den Berg

[noelani.vdberg@fabi.up.ac.za](mailto:noelani.vdberg@fabi.up.ac.za)

***Supplementary Material***


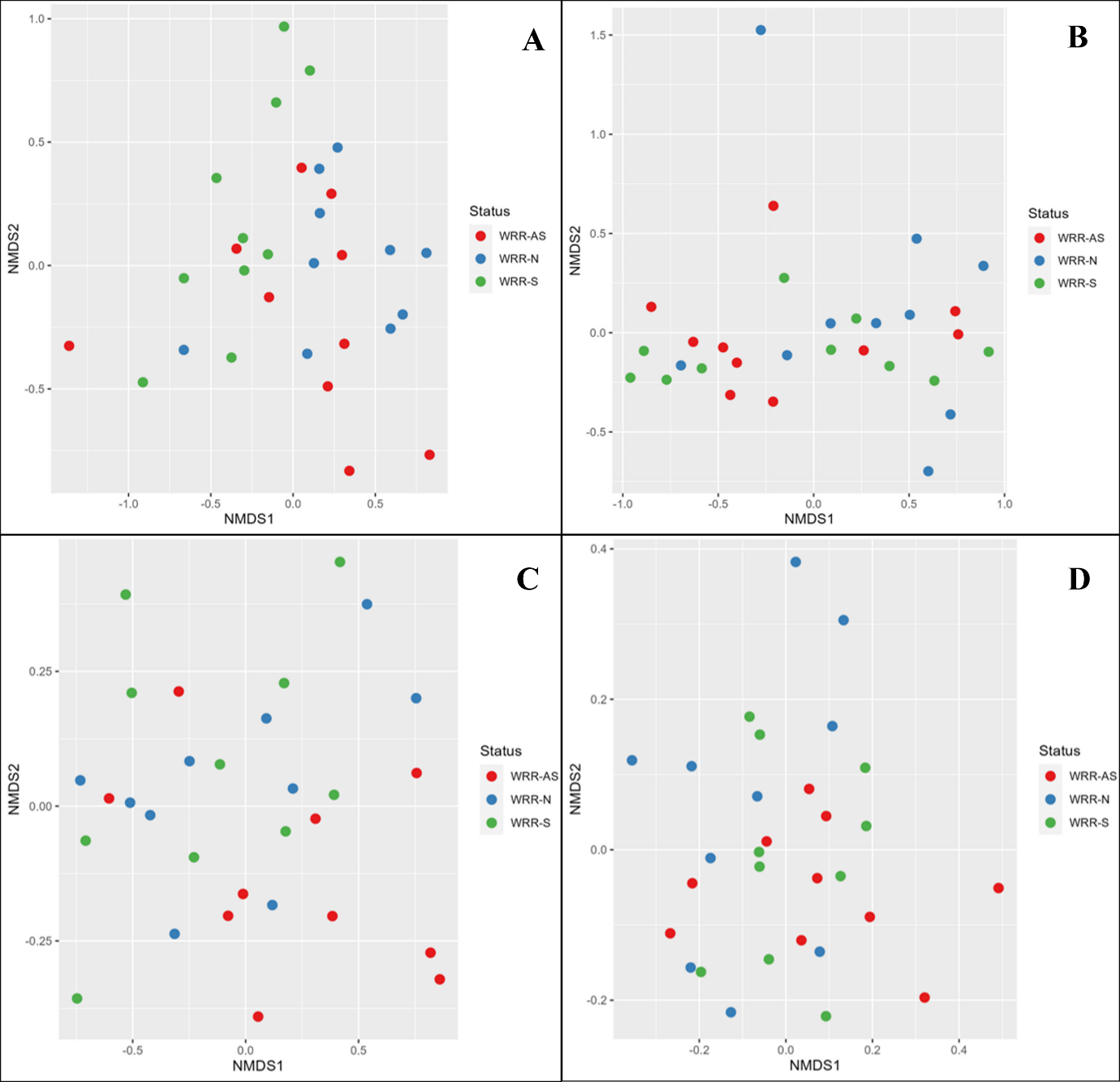


**Supplementary Figure 1: Beta diversity of the microbiome of bacterial and fungal communities in the avocado rhizosphere.** Bray-Curtis Beta diversity using Non-metric MultiDimensional Scaling (NMDS) of fungal and bacterial communities for **Orchard A (A**, fungal and **C**, bacteria) and **Orchard B** (**B**, fungal and **D**, bacteria) between white root rot asymptomatic (WRR-AS), non-infected (WRR-N), and symptomatic (WRR-S) rhizosphere soil samples. Horizontal bars within boxes represent the median. NMDS based on the Bray-Curtis distance matrix demonstrates the separation between WRR-S, WRR-N and WRR-AS.


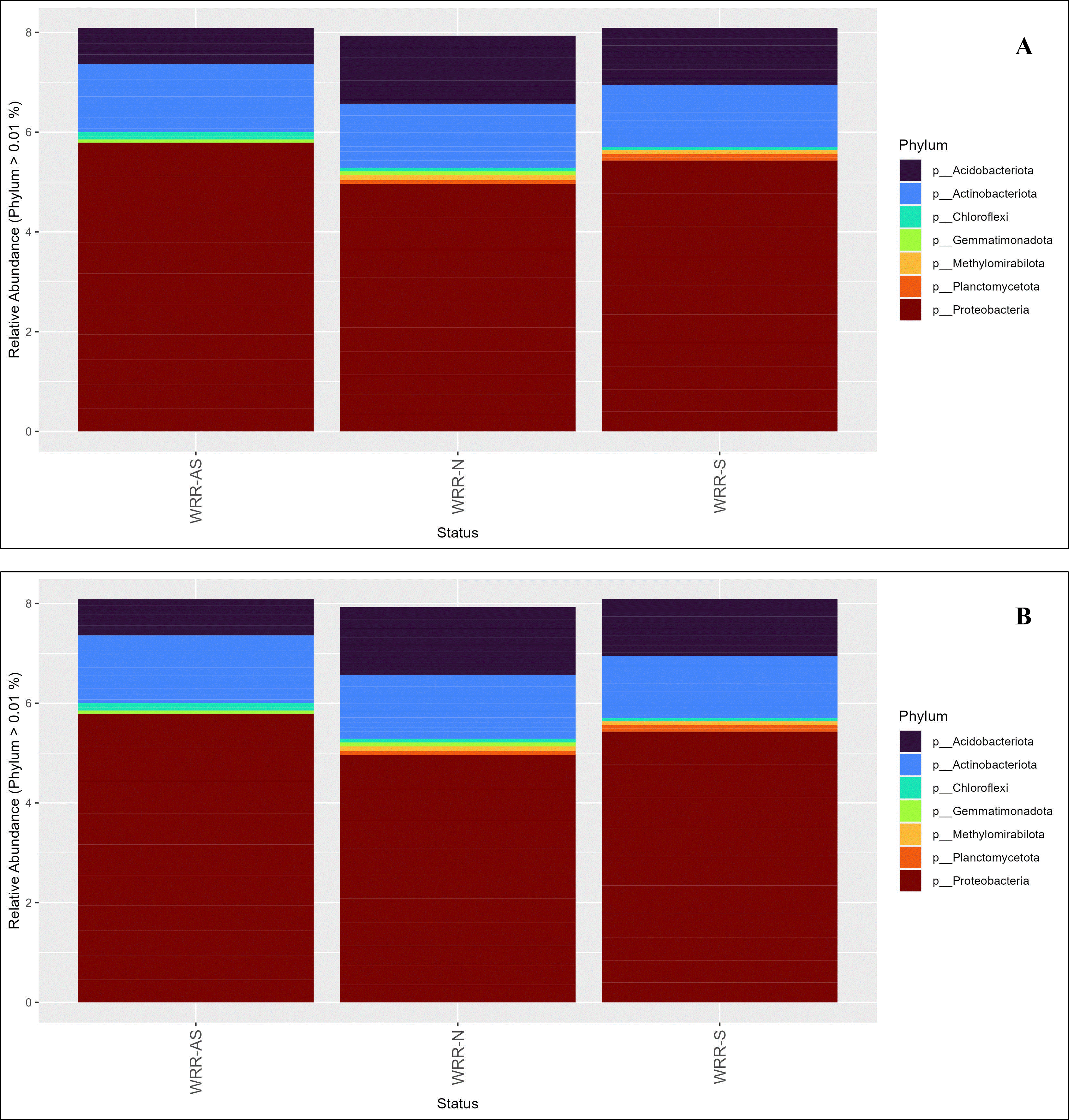


**Supplementary Figure 2: Relative abundance bar chart of the most abundant phylum of the bacterial community in the rhizosphere of samples collected in (A) Orchard A and (B) Orchard B.** Samples were collected from soil around white root rot asymptomatic (WRR-AS), white root rot non-infected (WRR-N), and white root rot symptomatic (WRR-S) trees. The most dominant genera above 0.01% were identified using 16S metabarcoding data.


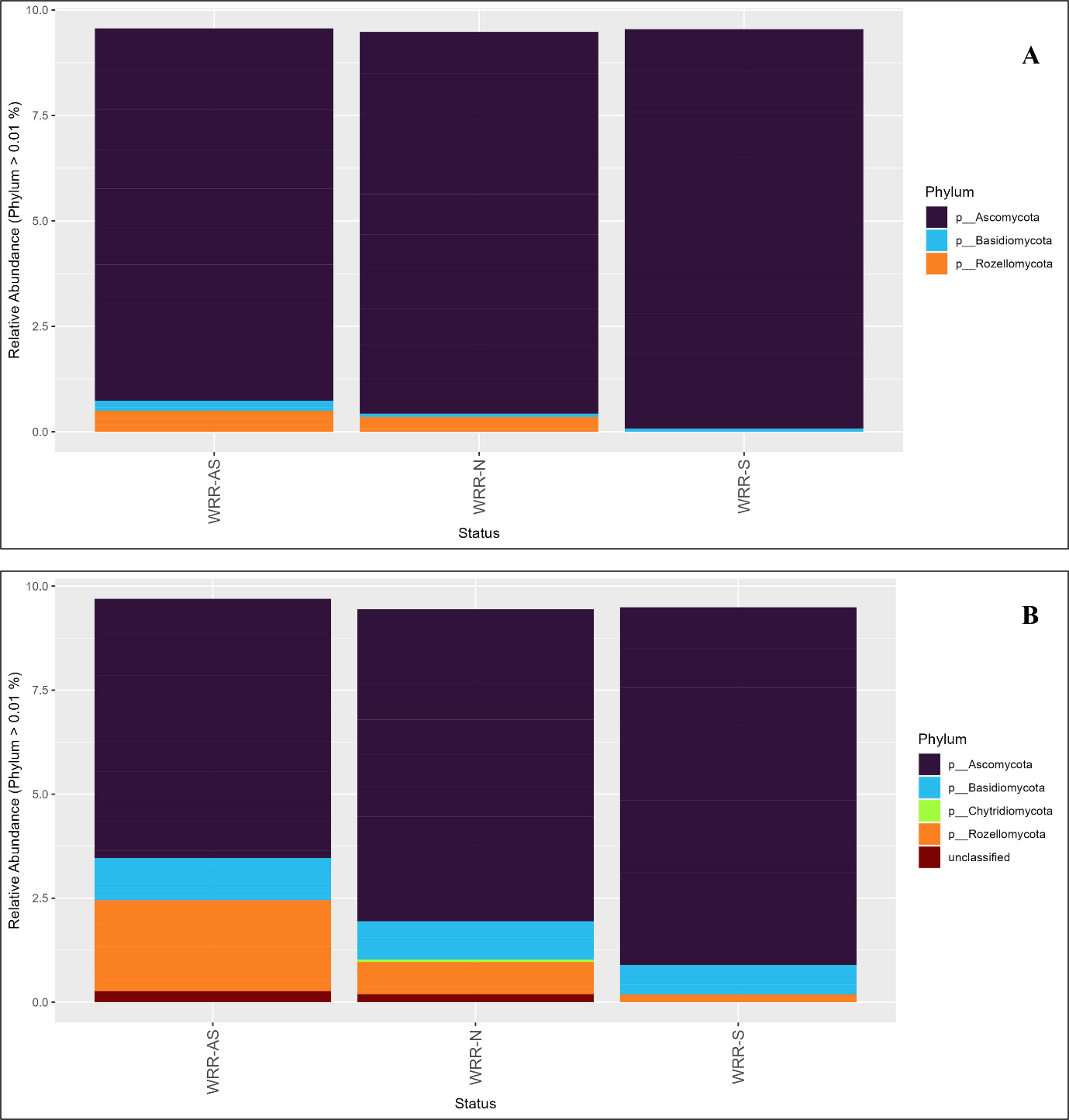


**Supplementary Figure 3: Relative abundance bar chart of the most abundant phylum of the fungal community in the rhizosphere of samples collected in (A) Orchard A and (B) Orchard B.** Samples were collected from soil around white root rot asymptomatic (WRR-AS), white root rot non-infected (WRR-N), and white root rot symptomatic (WRR-S) trees. The most dominant genera above 0.01% were identified using 16S metabarcoding data. Unclassified indicates the proportion of ASVs which could not be described to phylum level.


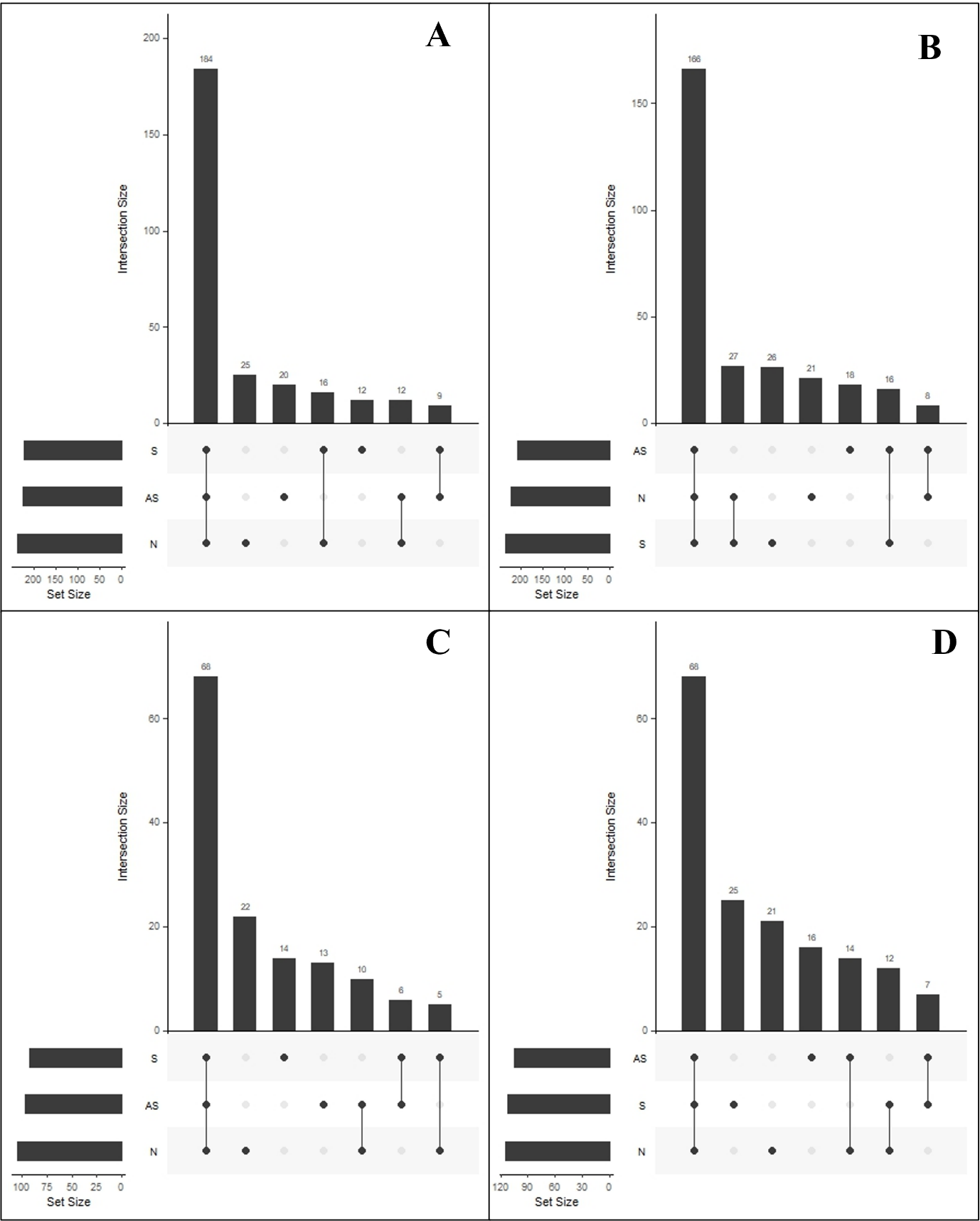


**Supplementary Figure 4: UpSet plot for the unique bacterial and fungal genera (relative abundance filtered at 0.001). (A)** Orchard A, bacterial genera, **(B)** Orchard B, bacterial genera, **(C)** Orchard A, fungal genera **(D)** Orchard B, fungal genera. The numbers at the top of each bar graph represents the number of genera either shared or unique. The connected dots with a line between AS (WRR-AS), N (WRR-N) and S (WRR-S) represent the shared genera and the single dots represents the unique genera for each category. The set size represents the total number of genera described in the particular data set.
